# Supplementary material for: The Russian war in Ukraine increased Ukrainian language use on social media
Source: Commun Psychol. 2024 Jan 10;2:1. doi: 10.1038/s44271-023-00045-6 (PMC11332000; doi:10.1038/s44271-023-00045-6)
Supplement: Supplementary file 1 — Reporting Summary [file 44271_2023_45_MOESM1_ESM.pdf]

nature portfolio

Corresponding author(s): Daniel Racek

Last updated by author(s): 13.11.2023

Reporting Summary

Nature Portfolio wishes to improve the reproducibility of the work that we publish. This form provides structure for consistency and transparency in reporting. For further information on Nature Portfolio policies, see our Editorial Policies and the Editorial Policy Checklist.

Statistics

For all statistical analyses, confirm that the following items are present in the figure legend, table legend, main text, or Methods section.

n/a	Confirmed	
☐	☒	The exact sample size (*n*) for each experimental group/condition, given as a discrete number and unit of measurement
☐	☒	A statement on whether measurements were taken from distinct samples or whether the same sample was measured repeatedly
☐	☒	The statistical test(s) used AND whether they are one- or two-sided<br>*Only common tests should be described solely by name; describe more complex techniques in the Methods section.*
☐	☒	A description of all covariates tested
☐	☒	A description of any assumptions or corrections, such as tests of normality and adjustment for multiple comparisons
☐	☒	A full description of the statistical parameters including central tendency (e.g. means) or other basic estimates (e.g. regression coefficient) AND variation (e.g. standard deviation) or associated estimates of uncertainty (e.g. confidence intervals)
☐	☒	For null hypothesis testing, the test statistic (e.g. F, t, r) with confidence intervals, effect sizes, degrees of freedom and P value noted<br>*Give P values as exact values whenever suitable.*
☒	☐	For Bayesian analysis, information on the choice of priors and Markov chain Monte Carlo settings
☒	☐	For hierarchical and complex designs, identification of the appropriate level for tests and full reporting of outcomes
☐	☒	Estimates of effect sizes (e.g. Cohen's *d*, Pearson's *r*), indicating how they were calculated

Our web collection on statistics for biologists contains articles on many of the points above.

Software and code

Policy information about availability of computer code

Data collection	Twitter API, through 1% stream (Standard API) & tweets/search/all endpoint (Research API)
Data analysis	All custom code used for analysis is available in an OSF repository. Link: https://osf.io/48sbc/

For manuscripts utilizing custom algorithms or software that are central to the research but not yet described in published literature, software must be made available to editors and reviewers. We strongly encourage code deposition in a community repository (e.g. GitHub). See the Nature Portfolio guidelines for submitting code & software for further information.

Data

Policy information about availability of data

All manuscripts must include a data availability statement. This statement should provide the following information, where applicable:

- Accession codes, unique identifiers, or web links for publicly available datasets
- A description of any restrictions on data availability
- For clinical datasets or third party data, please ensure that the statement adheres to our policy

The data is also available in the OSF repository. Link: https://osf.io/48sbc/

Human research participants

Policy information about studies involving human research participants and Sex and Gender in Research.

Reporting on sex and gender	Not possible, as demographic information was not collected.
Population characteristics	All Twitter users with at least one tweet with a Ukrainian geo-tag between 9th January 2020 and 12th October 2022. No further information on population characteristics (e.g. race, sex, gender, ...) available.
Recruitment	No recruitment, we collected all tweets with Ukrainian geo-information from 9th January 2020 to 12th October 2022.
Ethics oversight	Ethics commission of the faculty of mathematics, computer science and statistics at Ludwig-Maximilians-Universität (LMU) München

Note that full information on the approval of the study protocol must also be provided in the manuscript.

Field-specific reporting

Please select the one below that is the best fit for your research. If you are not sure, read the appropriate sections before making your selection.

☐ Life sciences ☒ Behavioural & social sciences ☐ Ecological, evolutionary & environmental sciences

For a reference copy of the document with all sections, see nature.com/documents/nr-reporting-summary-flat.pdf

Behavioural & social sciences study design

All studies must disclose on these points even when the disclosure is negative.

Study description	Study aimed to analyse tweets geolocated in the Ukraine before and during the Russia-Ukraine war (9th January 2020 to 12th October 2022). We specifically looked at how users changed their language over time.
Research sample	We included all Twitter users with at least one tweet with a Ukrainian geo-tag between 9th January 2020 and 12th October 2022.
Sampling strategy	All tweets available from the Twitter API given the spatial & temporal restrictions outlined here.
Data collection	We used the Twitter API, through the 1% stream (Standard API) and the tweets/search/all endpoint (Research API): 4,453,341 tweets from 62,712 users.
Timing	We collected the tweets between 9th January 2020 to 12th October 2022.
Data exclusions	~1.6 million tweets (~21,000 users) were removed during the data cleaning process.
Non-participation	Drop-out of users over time (as they stopped tweeting) was accounted for in the study design.
Randomization	No randomization was used.

Reporting for specific materials, systems and methods

We require information from authors about some types of materials, experimental systems and methods used in many studies. Here, indicate whether each material, system or method listed is relevant to your study. If you are not sure if a list item applies to your research, read the appropriate section before selecting a response.

Materials & experimental systems

n/a	Involved in the study	
☒	☐	Antibodies
☒	☐	Eukaryotic cell lines
☒	☐	Palaeontology and archaeology
☒	☐	Animals and other organisms
☒	☐	Clinical data
☒	☐	Dual use research of concern

Methods

n/a	Involved in the study	
☒	☐	ChIP-seq
☒	☐	Flow cytometry
☒	☐	MRI-based neuroimaging
