# Supplementary material for: The Russian war in Ukraine increased Ukrainian language use on social media
Source: Commun Psychol. 2024 Jan 10;2:1. doi: 10.1038/s44271-023-00045-6 (PMC11332000; doi:10.1038/s44271-023-00045-6)
Supplement: Supplementary file 3 — Supplementary Information [file 44271_2023_45_MOESM3_ESM.pdf]

Supplementary Material

Supplementary Notes 1: Topic Modelling

We conduct topic modelling on all Ukrainian, English, and Russian tweets after the pre-processing steps described in section 2.1 of the main paper (2,348,747 tweets). For each tweet text, we additionally remove any user mentions (via @), urls and all emojis.

In order to cluster the tweets across languages, multilingual topic modelling is required. Applying the clustering for each language individually would otherwise (most likely) result in topic clusters that cannot be perfectly matched between the different languages. This, in turn, would make any analysis much more difficult and potentially lead to incorrect conclusions. Here, we employ the Python implementation of BERTopic (Grootendorst, 2022), the most popular multilingual topic modelling in the research literature. The algorithm works as follows. First, all tweets are converted to numerical embeddings through a multilingual sentence embedding model (typically SBERT (Reimers and Gurevych, 2019)). Next, a dimensionality reduction is applied, which aims to retain the majority of the relevant information, while making the clustering computationally feasible. As a third step, a clustering algorithm is applied on the reduced feature space to find the topic clusters. Finally, through a bag-of-words approach over each cluster, topic representations (the most typical words) for each cluster are found.

In our implementation we opt for the following strategy. As a multilingual sentence embedding model, we choose the SBERT model *paraphrase-multilingual-MiniLM-L12-v2*, which is average-sized in terms of model parameters, but still performs well across sentence similarity tasks. For the dimensionality reduction, we employ the default UMAP (McInnes et al., 2018) (including default hyperparameters as suggested in BERTopic). We opt for the density-based clustering algorithm HDBSCAN (Campello et al., 2013). To reduce the amount of topic clusters, we choose a minimum cluster size of 11744, which corresponds to 0.5% of our tweets. Furthermore, we find the clustering to perform well (less noise) with $min\_samples = 3$ and $core\_distance = 10$. Finally, to improve our topic representations, we remove any stop words across all three languages (the stop word lists for the three languages are provided by *spacy* (Honnibal et al., 2020)).

Using this BERTopic configuration, we find a total of 33 topic clusters. Based on the representative words and corresponding tweets, we assign a suitable topic name for each of them. A full list is provided in Table 1. All representative words were translated retrospectively to English, hence duplicates can sometimes occur in the table. From the table we can see that there are two different topic clusters related to the war, with 250,030 and 91,949 tweets assigned respectively. The remaining ones are about a variety of different subjects such as family, romantic relationships, music, and COVID-19. We plot the topic distribution of the tweets over time for selected topics in Figure 1. The remaining plots can be found on the OSF. The figure shows that the topics not related to the war, plummet with its outbreak, but gradually return to pre-war levels after. The COVID-19 topic provides a good sanity check. It is most discussed with the first major outbreak in February and March 2020, and is also more prevalent during one of the heights of the pandemic in November 2021. With the mobilization of the Russian troops along the Ukrainian border and the subsequent outbreak of the war, the topic almost completely disappears from the tweets.

When it comes to the war topics, they seem to differ in what exactly users are discussing with respect to the war. The first war topic (topic #1), is more related to updates regarding the situation, asking for help, and supporting the people of Ukraine. The second war topic (topic #3) discusses war with respect to the political situation, power, and weapons. Both of the topics already appear in tweets before the outbreak of the war, which is not necessarily surprising, since the overall conflict between Ukraine and Russia has been going on for much longer. Nonetheless, as expected, the numbers considerably increase with the war. In Figure 2, we plot the war topics for each language over time. From there, we can see that before the outbreak of the war, both topics were generally discussed in both Ukrainian and Russian (but not in English), which changes thereafter. Then, the first war topic (updates, support, people), is mostly discussed in Ukrainian and English (with

a spike in English at the outbreak), whereas the second war topic (politics, weapons) mostly in Ukrainian. Notably, the Russian tweet numbers about the war almost remain on pre-war levels (with the exception of the outbreak).

Supplementary Table 1: Topic Clusters

#	Topic Name	Representative Words	# of Tweets
1	War - help, support, people, updates	'ukraine', 'russian', 'russia', 'ukraine', 'ukraine', 'ukrainian', 'kyiv', 'ukraine', 'war', 'people', 'russians', 'ukraine', 'rf', 'kyiv', 'ukraine', 'putin', 'russiaisaterroriststate', 'ukraine', 'ukraine', 'ukrainians', 'russia', 'russia', 'kyiv', 'krym', 'ukrainians', 'russia', 'russia', 'ukrainians', 'glory', 'stoprussia', 'kharkiv', 'ukrainian', 'standwithukraine', 'kiev', 'russia', 'putin', 'city', 'russiaisaterroriststate', 'world', 'lviv', 'today', 'poroshenko', 'military', 'country', 'people', 'people', 'day', 'amp', 'support', 'kiev'	250,030
2	Greetings, hello	'tomorrow', 'sleep', 'sleep', 'today', 'morning', 'morning', 'sleep', 'morning', 'home', 'good', 'night', 'good', 'hours', 'morning', 'morning', 'night', 'sleep', 'hours', 'day', '00', 'good', 'week', 'home', 'night', 'night', 'evening', 'evening', '10', 'minutes', 'hours', 'home', 'yesterday', 'hours', 'slept', 'days', 'yesterday', '30', 'what', 'woke up', 'morning', 'apartment', 'good', 'home', 'night', 'days', 'weeks', 'sleep', 'morning', 'week', 'Monday'	91,949
3	War - politics, power, weapons	'wars', 'war', 'president', 'president', 'people', 'people', 'war', 'usa', 'death', 'sho', 'wars', 'people', 'war', 'war', 'army', 'court', 'country', 'country', 'country', 'army', 'law', 'law', 'missiles', 'country', 'country', 'missiles', 'election', 'ze', 'countries', 'generally', 'don', 'authorities', 'trump', 'army', 'remember', 'life', 'rights', 'authorities', 'think', 'people', 'like', 'country', 'right', 'know', 'nobody', 'military', 'question', 'country', 'president', 'someone'	90,021
4	Emotional (with swearing)	'fuck', 'fuck off', 'bitch', 'fuck', 'sho', 'slovo', 'fuck', 'fuck', 'cunt', 'whore', 'dick', 'giza', 'cunt', 'fuck', 'yell', 'go', 'god', 'nah', 'bees', 'gt', 'ahhhh', 'this', 'la', 'shit', 'fuck', 'mosquito', 'gg', 'love', 'go', 'everything', 'horror', 'f**k', 'horror', 'mosquitoes', 'gross', 'zee', 'shit', 'cunt', 'mosquitoes', 'mosquitoes', 'cunt', 'aaaa', 'norm', 'may', 'mi', 'ahahahahaha', 'high', 'god', 'birds', 'birdie', 'birdies'	78,742
5	Family, school	'mum', 'children', 'years', 'children', '10', 'children', 'mother', 'children', 'school', '20', '30', 'parents', 'sho', 'brother', 'parents', '18', 'sister', 'parents', 'study', 'grandfather', 'class', 'child', 'schools', 'know', 'school', '25', '15', 'school', 'mum', 'boy', 'school', 'dad', 'dad', 'life', 'mums', 'fuck', '16', 'people', 'diploma', 'mum', 'grandfather', 'parents', 'today', '17', 'father', 'mother', 'mums', 'class', '40', 'people'	62,203
6	Feelings	'know', 'can', 'fuck', 'what', 'feel', 'understand', 'life', 'life', 'emotions', 'fuck', 'understand', 'fuck', 'people', 'don', 'do', 'bitch', 'understood', 'no', 'generally', 'emotions', 'people', 'feel', 'have', 'feeling', 'thought', 'god', 'knew', 'live', 'think', 'feel', 'understood', 'live', 'do', 'want', 'fuck', 'shit', 'do', 'strongly', 'want', 'emotions', 'do', 'no', 'did', 'emotions', 'okay', 'time', 'love', 'huh', 'loneliness'	55,066
7	Romantic relationships	'sex', 'girls', 'woman', 'woman', 'women', 'women', 'women', 'women', 'girls', 'men', 'men', 'men', 'porn', 'girl', 'girl', 'men', 'show', 'girls', 'wedding', 'girl', 'men', 'married', 'girl', 'married', 'girls', 'sex', 'sex', 'women', 'gay', 'men', 'man', 'man', 'know', 'sexymonday', 'lgbt', 'woman', 'sex', 'wife', 'sex', 'girl', 'wife', 'love', 'sex', 'girls', 'men', 'man', 'husband', 'people', 'men', 'think', 'girls'	47,146
8	Food	'eat', 'kg', 'tasty', 'delicious', 'tasty', 'soup', 'cake', 'salad', 'shower', 'breakfast', 'lunch', 'cook', 'ate', 'recipe', 'like', 'kitchen', 'cook', 'taste', 'eat', 'food', 'what', 'chocolate', 'butter', 'bread', 'breakfast', 'today', 'meat', 'toilet', 'taste', 'meat', 'food', 'food', 'dinner', 'food', 'ate', 'food', 'delicious', 'food', 'mum', 'potatoes', 'cheese', 'menu', 'cheese', 'kitchen', 'eat', 'know', 'bread', 'weight', 'dinner', 'sauce'	45,166
9	Transportation	'subway', 'car', 'city', 'car', 'taxi', 'cities', 'car', 'city', 'taxi', 'flowers', 'roads', 'gas', 'driver', 'flowers', 'city', 'tesla', 'taxi driver', 'bus', 'sho', 'village', '007', 'train', 'cities', 'people', 'streets', 'cars', 'km', '38', 'city', 'bike', 'people', 'tesla', '10', 'go', 'transport', 'tree', '22', 'village', 'train', 'tram', 'car', '00', 'trees', 'car', 'forest', 'UAH', 'villages', 'driver', 'deteling', 'ride'	42,911
10	Art and photos	'photo', 'art', 'hair', 'hairs', 'photos', 'artwork', 'nft', 'color', 'artist', 'photo', 'call', 'like', 'nftcommunity', 'hair', 'artist', 'photos', 'photo', 'black', 'painting', 'new', 'photo', 'collection', 'art', 'drawing', 'photography', 'photos', 'beautiful', 'hair', 'love', 'photo', 'sketch', 'photoshop', 'colors', 'colors', 'digitalart', 'abstract', 'color', 'picture', 'illustration', 'draw', 'photographer', 'film', 'camera', 'nfts', 'portrait', 'white', 'available', 'artists', 'nftart'	40,873
11	Music	'album', 'song', 'song', 'concert', 'songs', 'songs', 'song', 'music', 'song', 'song', 'voice', 'music', 'music', 'listen', 'music', 'music', 'listen', 'sound', 'album', 'listen', 'listen', 'dance', 'listen', 'songs', 'songs', 'songs', 'music', 'headphones', 'rock', 'radio', 'music', 'track', 'dance', 'sound', 'love', 'album', 'know', 'album', 'listen', 'love', 'soundcloud', 'sing', 'sound', 'what', 'hear', 'dance', 'sing', 'headphones', 'ears', 'dance'	35,889

12	Money	'pennies', 'UAH', 'money', '100', 'money', 'pennies', 'money', '50', 'buy', 'price', 'shop', 'buy', 'hryvnia', '200', '10', 'price', 'bought', '500', 'expensive', 'bank', 'pay', '1000', '300', 'budget', 'sho', 'bought', 'taxes', '20', 'prices', 'pay', 'cards', 'salary', 'million', 'people', 't9', 'bank', '73', '80', 'people', 'card', 'credit', 'cheaper', 'salary', '000', 'buy', '90', '30', 'banks', 'price', 'bought'	35,744
13	Weather	'rain', 'weather', 'spring', 'snow', 'cold', 'summer', 'snow', 'winter', 'sun', 'summer', 'rain', 'temperature', 'sun', 'warm', 'autumn', 'light', 'summer', 'hot', 'weather', 'winter', 'sun', 'autumn', 'wind', 'street', 'snow', 'summer', 'street', 'summer', 'wind', 'winter', 'today', 'light', 'spring', 'rain', 'temperature', 'winter', 'winter', 'snow', 'light', 'heat', 'summer', 'snow', 'love', 'winter', 'cold', 'air', 'summer', 'hot', 'fog', 'spring'	34,078
14	Social media, Twitter	'twitter', 'tweet', 'tweet', 'twitter', 'twitterer', 'twitter', 'twitter', 'tweets', 'tweeters', 'twitter', 'safeairliftukraine', 'deaths', 'tweets', 'assistance', 'defend', 'innocent', 'provide', 'humanitarian', 'civilian', 'needs', 'weapons', 'stopputin', 'lamp', 'twitter', 'tweets', 'stop', 'pride', 'tweet', 'putin', 'joy', 'instagram', 'prideandjoy', 'twitter', 'facebook', 'amp', 'tweets', 'social', 'lightingdesign', 'lamps', 'instagram', 'ukraine', 'facebook', 'tweets', 'interiordesign', 'tweet', 'tweeting', 'tweeter', 'show', 'tweeter', 'people', 'exclusive'	32,462
15	Thoughts about life	'life', 'life', 'people', 'no', 'people', 'life', 'nobody', 'live', 'life', 'don't', 'live', 'no', 'generally', 'main', 'person', 'what', 'person', 'life', 'main thing', 'people', 'person', 'impossible', 'do', 'argument', 'alive', 'normal', 'people', 'works', 'live', 'people', 'no way', 'better', 'worth', 'works', 'mistakes', 'think', 'no', 'problems', 'fuck', 'sense', 'do', 'opinion', 'question', 'ok', 'live', 'mistakes', 'want', 'problem', 'hard', 'motivation'	30,927
16	Films and videos	'video', 'video', 'film', 'film', 'video', 'season', 'youtube', 'series', 'film', 'series', 'channel', 'watch', 'film', 'show', 'anime', 'tiktok', 'series', 'films', 'films', 'series', 'watch', 'watch', 'season', 'film', 'watch', 'film', 'film', 'watch', 'watch', '10', 'watch', 'character', 'films', 'watched', 'watched', 'clip', 'theatre', 'series', 'series', 'plot', 'series', 'youtube', 'series', 'love', 'videos', 'films', 'channel', 'series', 'show', 'character'	29.989
17	Phones	'phone', 'apple', 'iphone', 'iPhone', 'internet', 'internet', 'phone', 'ios', 'phone', 'phone', 'online', 'site', 'account', 'content', 'app', 'phone', 'pro', 'chat', 'phone', 'message', 'android', 'neverlock', 'app', 'lock', 'number', 'link', 'usb', 'model', 'bundle', 'macbook', 'price', '10', 'availability', 'sms', 'mail', 'internet', 'samsung', 'show', 'works', 'subscribers', 'ipad', 'app', 'account', 'account', 'internet', 'colour', 'code', 'smartphone', 'new', 'condition'	27,236
18	Congratulations	'thank you', 'thank you', 'thanks', 'congratulations', 'big', 'greetings', 'support', 'congratulations', 'congratulations', 'hello', 'hello', 'thank you', 'nice', 'congrats', 'work', 'appreciate', 'sincerely', 'support', 'huge', 'sir', 'dear', 'grateful', 'great', 'words', 'nice', 'in return', 'job', 'lady', 'friend', 'congratulations', 'hello', 'great', 'wow', 'thank you', 'lot', 'support', 'words', 'welcome', 'hear', 'compliment', 'love', 'support', 'compliment', 'amazing', 'friend', 'thank you', 'grateful', 'ohhh', 'works', 'brother'	26,645
19	Football (soccer)	['books', 'read', 'book', 'read', 'write', 'write', 'read', 'book', 'write', 'books', 'read', 'write', 'write', 'write', 'book', 'read', 'book', 'write', 'write', 'write', 'author', 'write', 'write', 'read', 'read', 'book', 'text', 'wrote', 'book', 'write', 'booklet', 'read', 'read', 'read', 'sh', 'author', 'wrote', 'know', 'written', 'read', 'wrote', 'write', 'can', 'read', 'stop', 'ff', 'write', 'read', 'write', 'read', 'read', 'writes']	25,644
20	Books and reading	'books', 'read', 'book', 'read', 'write', 'write', 'read', 'book', 'write', 'books', 'read', 'write', 'write', 'write', 'book', 'read', 'book', 'write', 'write', 'write', 'author', 'write', 'write', 'read', 'read', 'book', 'text', 'wrote', 'book', 'write', 'booklet', 'read', 'read', 'read', 'sh', 'author', 'wrote', 'know', 'written', 'read', 'wrote', 'write', 'can', 'read', 'stop', 'ff', 'write', 'read', 'write', 'read', 'read', 'writes'	21,758
21	Religion	'god', 'god', 'god', 'church', 'lord', 'christ', 'holy', 'church', 'give', 'rise', 'lord', 'church', 'jesus', 'god', 'heaven', 'god', 'miracle', 'church', 'queen', 'king', 'church', 'queen', 'kingdom', 'pope', 'church', 'pekli', 'grih', 'temple', 'hell', 'inferno', 'pray', 'christ', 'gods', 'heavenly', 'jesus', 'easter', 'king', 'holy', 'paradise', 'glory', 'people', 'petro', 'prince', 'hell', 'issus', 'sho', 'lord', 'holy', 'crown'	21,016
22	Holiday season	'year', '2020', '2021', 'year', 'year', '2022', 'holy', 'new', 'years', 'christmas', 'gift', 'year', 'gift', 'novy', 'holiday', 'new', 'new', 'holy', 'year', 'gift', 'presents', 'presents', 'new', 'holiday', 'new', 'season', '2019', 'happy', 'march', 'february', 'luteo', 'sichnya', 'yalinku', 'beresnya', 'groudnya', 'holy', '2018', 'svetkuvat', 'festivities', 'party', '2014', 'calendar', '24', 'ridzdom', 'ridztwo', 'today', 'january', 'merry', '14', 'december'	18,657
23	Drinking and alcohol	'beer', 'wine', 'beer', 'beer', 'drink', 'wine', 'piti', 'alcohol', 'drink', 'beer', 'beer', 'drink', 'saw', 'pyla', 'plyashka', 'vipiti', 'bottle', 'viski', 'whisky', 'whiskey', 'pivka', 'alcohol', 'vodka', 'pivko', 'drank', 'vodka', 'pachashka', 'champagne', 'alcohol', 'bottle', 'piv', 'sho', 'wine', 'juice', 'vip', 'pili', 'vipila', 'drunk', 'drunk', 'vodka', 'today', 'drink', 'sik', 'beer', 'wine', 'cocktail', 'vipiv', 'bottle', 'champagne', 'beerloga_drinks', 'birlogamagazinrazlivnonopivo', 'drank', 'birlogamakeevka', 'drunk', 'glass'	18,382

24	Swearing	'sho', 'bitch', 'fuck', 'fuck', 'fuck', 'fuck', 'what', 'fuck', 'fuck', 'fuck', 'god', 'deeds', 'you do', 'what', 'you do', 'fuck', 'wow', 'you tell', 'ban', 'nye', 'ouch', 'you got', 'you call', 'go', 'fuck', 'say', 'fool', 'tell', 'fuck', 'cunt', 'fuck', 'go', 'fuck', 'tell', 'tell', 'panel', 'shob', 'stupid', 'dey', 'shit', 'mean', 'keep up', 'you know', 'yake', 'fuck', 'you know', 'shit', 'shit', 'shit', 'hello', 'fuck'	17,992
25	Love	'love', 'love', 'love', 'love', 'love', 'love', 'love', 'kohannya', 'adore', 'like', 'love', 'love', 'adore', 'like', 'like', 'strongly', 'lubovi', 'fell in love', 'love', 'love', 'love', 'like', 'romance', 'love', 'beloved', 'love', 'love', 'fall in love', 'person', 'fell in love', 'people', 'know', 'favourite', 'life', 'loved', 'loved', 'beloved', 'lubish', 'romantic', 'friend', 'life', 'love', 'loved', 'loved', 'in love', 'favourite', 'ulyubleniye', 'liked', 'ulyubeleniye', 'life', 'love', 'ulyubleniye', 'don', 'heart', 'kohayu', 'person'	17,069
26	Compliments	'beautiful', 'pretty', 'cool', 'beautiful', 'beauty', 'beauty', 'gorgeous', 'lovely', 'lovely', 'gorgeous', 'gorgeous', 'wow', 'cool', 'beauty', 'beautiful', 'beautiful', 'beauty', 'beauty', 'super', 'good', 'looks', 'wonder', 'handsome', 'gorgeous', 'amazing', 'beautiful', 'garni', 'beauty', 'wow', 'cool', 'beauty', 'neymovirno', 'beauty', 'handsome', 'classy', 'look', 'beautiful', 'awesome', 'beautiful', 'beautiful', 'not bad', 'genial', 'good', 'genial', 'nice', 'beautiful', 'god', 'looks', 'beautiful'	16,909
27	Talking about other people	'name', 'name', 'what', 'dude', 'guy', 'surname', 'think', 'name', 'man', 'name', 'know', 'name', 'said', 'man', 'knows', 'dick', 'knows', 'man', 'surname', 'name', 'generally', 'name', 'man', 'interesting', 'says', 'does', 'man', 'like', 'wants', 'went', 'man', 'fuck', 'did', 'don', 'names', 'does', 'name', 'wants', 'fuck', 'hope', 'name', 'really', 'fuck', 'name', 'did', 'dey', 'know', 'guy', 'wanted', 'surname'	16,167
28	Health and doctors	'health', 'doctor', 'hospital', 'doctor', 'recovery', 'doctor', 'doctor's', 'doctors', 'doctors', 'doctors', 'health', 'healthcare', 'cough', 'doctor', 'health', 'breathe', 'hospital', 'breathing', 'emergency', 'doctors', 'medicine', 'hospital', 'doctors', 'hospital', 'test', 'what', 'COVID', 'hospitals', 'diagnosis', 'doctor', 'people', 'doctors', 'nurse', 'temperature', 'medicine', 'doctors', 'diagnosis', 'disease', 'people', 'hospital', 'syndrome', 'know', 'patients', 'treatment', 'patients', 'doctor', 'doctor's', 'sick', 'sickness', 'sicknesses', 'ambulance'	15,077
29	COVID-19	'COVID', 'coronavirus', '19', 'COVID19', 'coronavirus', 'cases', 'coronavirus', 'vaccination', 'vaccines', 'vaccine', 'vaccine', 'vaccine', 'news', 'coronavirus', 'day', 'new', 'virus', 'coronavirus', 'virus', 'recorded', 'quarantine', 'disease', 'vaccination', 'brain', 'ukraine', 'people', 'vaccines', 'vaccination', 'coronavirus', 'vaccination', 'confirmed', 'news', 'vaccination', 'condition', 'dose', 'coronavirus', 'vaccine', 'vaccines', 'virus', 'ukraine', 'patients', 'test', 'disease', 'number', 'people', 'pfizer', 'coronavirus', 'virus', 'read', 'virus'	13,533
30	Cats	'cat', 'cat', 'cat', 'cat', 'cat', 'cats', 'cats', 'cats', 'cats', 'cats', 'cats', 'cats', 'cats', 'cats', 'cat', 'cat', 'cats', 'cats', 'cat', 'cat', 'cats', 'cats', 'cats', 'cats', 'cats', 'cats', 'cat', 'cat', 'cat', 'cats', 'food', 'muzzle', 'animal', 'kitten', 'love', 'what', 'cats', 'cat', 'kitty', 'cat', 'cat, 'kitten', 'kittens', 'today', 'kittens', 'kittens', 'kitty', 'home', 'kittens', 'dogs', 'cat', 'sevastopol'	13,366
31	Sea, lakes, water	'sea', 'water', 'water', 'sea', 'water', 'beach', 'water', 'lake', 'dnipro', 'sea', 'sea', 'beach', 'beach', 'water', 'ocean', 'ocean', 'water', 'lakes', 'beaches', 'swim', 'water', 'beach', 'swimsuit', 'shore', 'shore', 'water', 'water', 'pool', 'swim', 'river', 'melekino', 'sea', 'pool', 'swim', 'sea', 'promenade', 'marina', 'today', 'beach', 'river', 'waves', 'shore', 'swimming', 'swim', 'island', 'promenade', 'beach', 'waves', 'what', 'ship', 'river', 'underwater'	12,732
32	Work	'work', 'work', 'work', 'work', 'work', 'work', 'work', 'work', 'work', 'work', 'interview', 'work', ' office', 'interview', 'working', 'business', 'boss', 'resume', 'office', 'business', 'job', 'working', 'companies', 'office', 'companies' , 'office', 'company', 'manager', 'boss', 'sho', 'interview', 'business', 'you work', 'interview', 'company', 'company', 'you work', ' interview', 'people', 'interviews', 'company', 'people', 'do', 'boss', 'love', 'know', 'can', 'time', 'superiors', 'director'	12,208
33	Non-alcoholic drinks, coffee	'coffee', 'tea', 'coffee', 'coffee', 'coffee', 'coffee', 'coffee', 'tea', 'cafe', 'coffee', 'fridge', 'drink', 'tea', 'restaurant', 'drink', 'drink', 'cups', 'cup', 'café', 'morning', 'cup', 'tea', 'morning', 'drink', 'fridge', 'kettle', 'café', 'milk', 'shop', 'drink', 'cappuccino', 'morning', 'coffee shop', 'like', 'tea', 'drank', 'today', 'fridge', 'starts', 'morning', 'coffee photos', 'watermelons', 'jo', 'tea', 'cup', 'tasty', 'barista', 'cocoa', 'watermelon', 'starts'	11,760

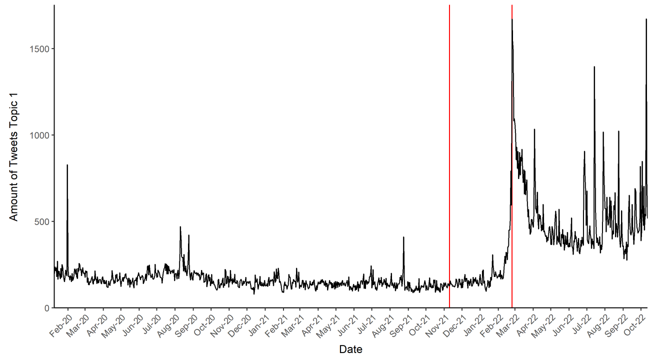

(a) War - updates, people, help, support

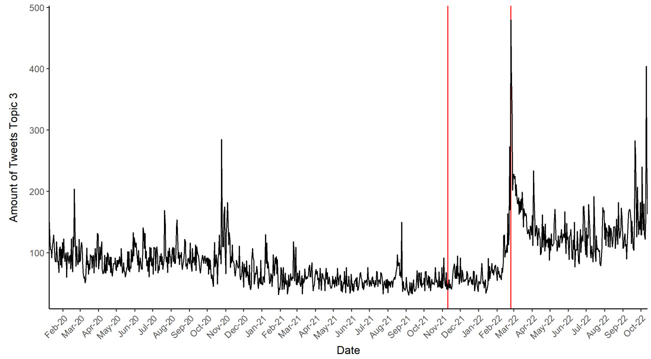

(b) War - politics, power, weapons

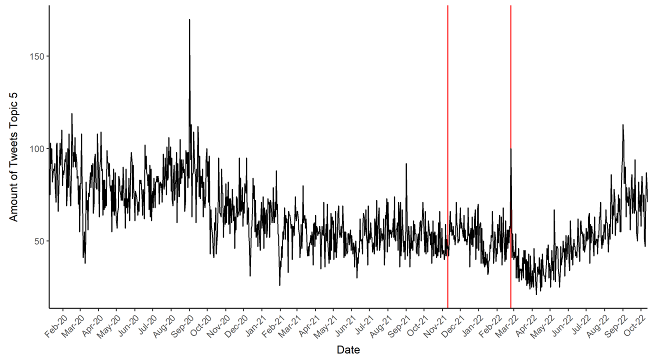

(c) Family, school

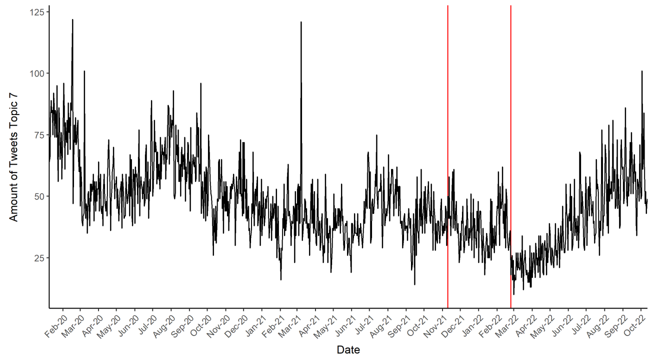

(d) Romantic relationships

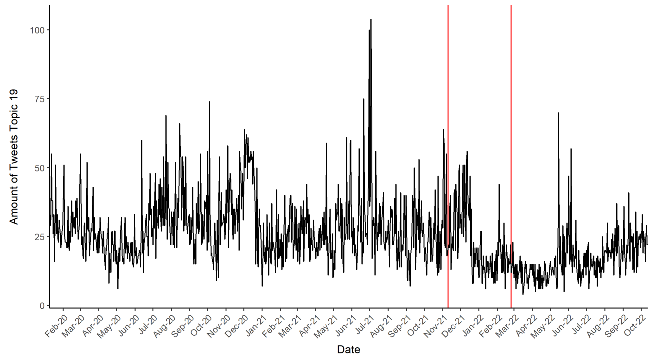

(e) Football (soccer)

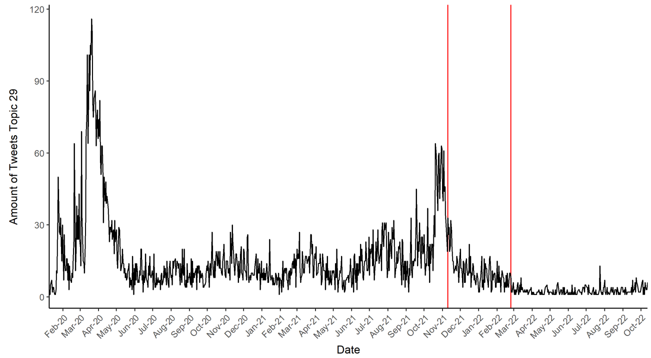

(f) COVID-19

Supplementary Figure 1: Topic distribution of tweets over time (daily). The first vertical line denotes the mobilization of the Russian troops along the Ukrainian border. The second line denotes the outbreak of the war.

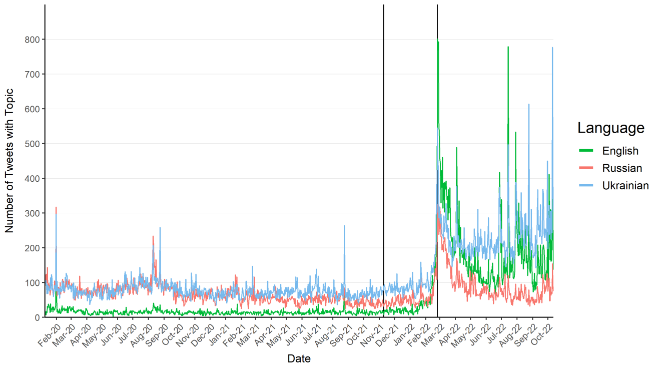

(a) War - updates, people, help, support

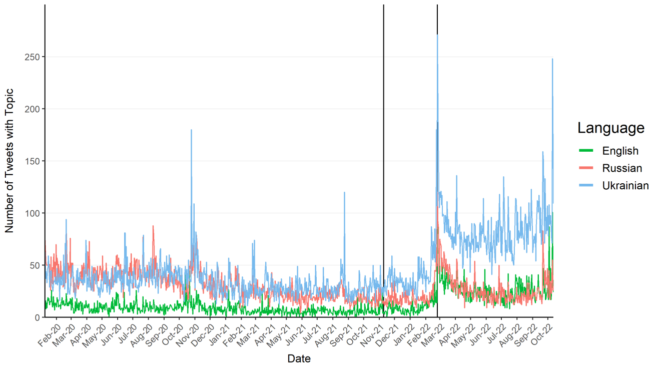

(b) War - politics, power, weapons

Supplementary Figure 2: War topic distribution per language over time (daily). The first vertical line denotes the mobilization of the Russian troops along the Ukrainian border. The second line denotes the outbreak of the war.

Supplementary Notes 2: Alternative Modelling Strategies

Supplementary Notes 2.1: Factor Smooths

One of the limitations of our modelling specification in the main paper (for both tweet and language model) is, that for simplification purposes, we assume time-constant random intercepts for each user, and a smooth global trend. The latter, which changes over time, should capture behavioural changes across all users (see section 4.4 and 4.5 in the main paper). Naturally, this by definition "averages" user behaviour over time and it does not allow us to differentiate between users that exhibit a change in behaviour that deviates from this average.

Instead, factor smooths, which can model behavioural changes for each user separately over time (and a potential deviation from the average trend) would be a more appropriate choice. However, our simplification is necessary, as, to the best of our knowledge, this would be computationally unfeasible to estimate given the size of our dataset. Additionally, our chosen specification with random intercepts should be able to approximate any global changes in behaviour (how does the average user change over time?) reasonably well. This comes at the cost of a loss of information with respect to changes of individual users. We try to make up for this loss in information with our descriptive plots in Figure 5 of the main paper. Subsequently, we will discuss the computational difficulties of estimation and show that we are able to approximate and capture the global behavioural changes well with our chosen model specification.

The R package *mgcv* offers two ways to implement these factor smooths. The first variant using the option $bs =$ "sz" would force the main smooth (in our case the global time trend) to capture as much of the effect as possible, hence the preferred variant. The second option using $bs =$ "fs" would treat each user factor smooth as random (but without the main effect constraint), which speeds up the estimation process. We tried to estimate both variants for both tweet and language model with the full set of users (13,643), as well as a random subset (1,500) with a compute node offering up to 170GB of RAM. For the full set of users, the memory limit was exceeded for both variants and both models. For the first variant, it was even exceeded for the smaller subset. Hence, estimation was not possible at all. For the second variant ($bs =$ "fs"), estimation was only possible for the smaller subset, and there only for the language model (42 GB of memory required), which includes fewer smoothing components, as for each language pair the estimation is carried out separately. Hence, we only re-estimated the language model(s), with the random subset of 1,500 users using the random curves ($bs =$ "fs"). This means, we again model $X_{t,u} \sim Binomial(n_{t,u}, \pi_{t,u})$, but define the probability to tweet in one language over another as

$$\pi_{t,u} = f(\mu + s(t) + W_u(t)),$$

where the $W_u(t)$ are now modelled as random curves that change over time.

Figure 3 shows the behavioural effects (global time trend) for the three language pairs (a) for the factor smooth model with the random subsample of 1,500 users, (b) for the random intercept model (specification as in main paper) with the same random subsample of 1,500 users, (c) for the random intercept model with all users as also reported in the main paper. By comparing the three plots we can observe that the general trends between factor smooth and random intercept model remain roughly the same (with small differences in the UK over EN language pair). In fact, the magnitudes of the coefficients are also highly similar, even though they were only estimated using around a tenth of the original user sample (small differences between the two random intercept models are also apparent). As expected, the confidence intervals are larger with less users and larger when using factor smooths. Overall, based on those results, we argue that the behavioural effects reported in the main paper, using the simplified model with random intercepts, seem to be well approximated.

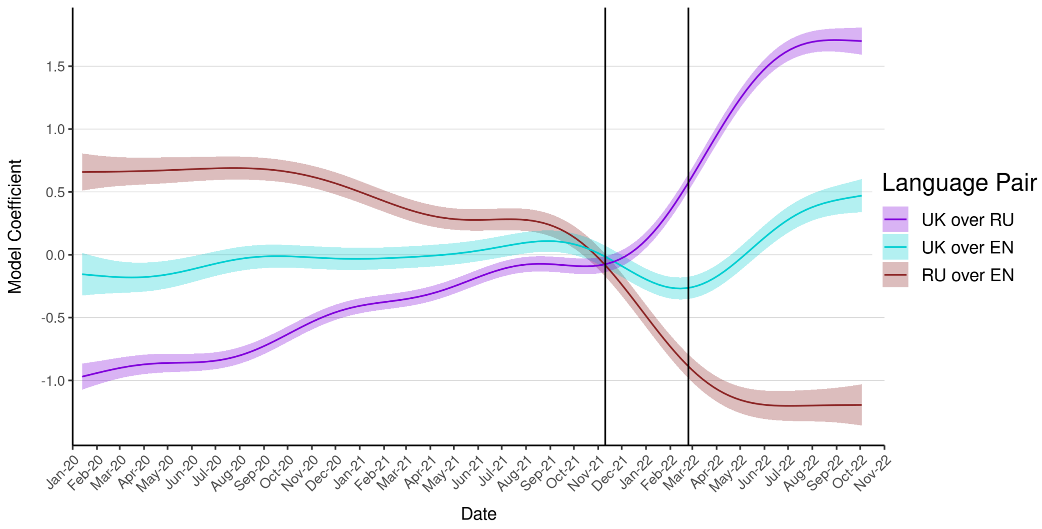

(a) Factor smooth specification. Random subsample of users (1,500) .

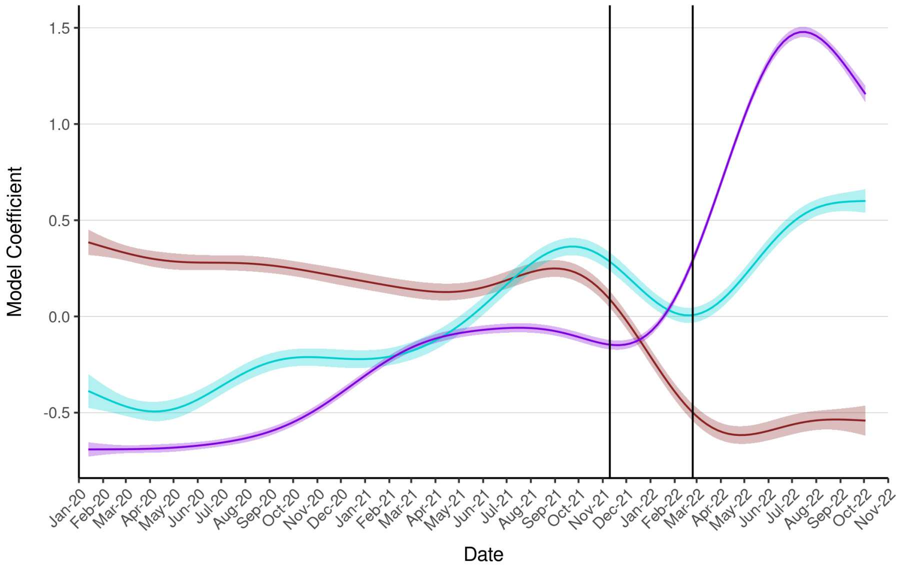

(b) Specification as in main paper. Random subsample of users (1,500).

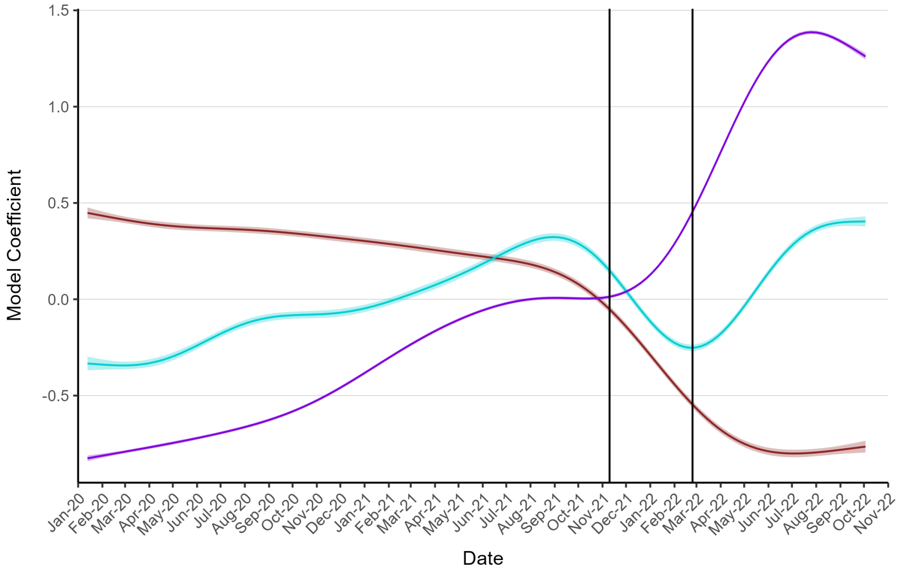

(c) Specification as in main paper. Full user sample (13,643).

Supplementary Figure 3: Behavioural effects. The graphs report the fitted global trend over all users in each week for all three language pairs. The shaded area depicts the 95% confidence interval of the fitted effect. The first vertical line denotes the mobilization of the Russian troops along the Ukrainian border. The second line denotes the outbreak of the war.

Supplementary Notes 2.2: Regression Discontinuity Design

Particularly in economics, regression discontinuity (RD) designs are commonly employed to draw causal inferences, as they only require "mild" assumptions compared to many other non-experimental approaches (see e.g. Lee and Lemieux (2010) for a thorough introduction). However, as discussed in Hausman and Rapson (2018), when using time as a running variable with a treatment date as the threshold, one deviates from the classic cross-sectional RD framework, often referred to as regression discontinuity in time (RDiT). One of the major limitations of such RDiT designs is that time-varying treatment effects violate the RDiT assumptions and are untestable. Instead, the researcher must assume how the treatment effect evolves over time, or control units are required (Hausman and Rapson, 2018, p.543). This becomes particularly difficult when wanting to differentiate between short- and long-term effects of the treatment. Another issue arises when using higher-order polynomial fits, as one often introduces a bias into the estimates due to the treatment cutoff. Following this design, in our setting, the outbreak of the war is the treatment. We assume (and show) that the treatment effect of the war on tweet activity and language choice is varying in time (e.g. jump in number of English tweets with outbreak of the war and gradual decrease thereafter) and partially already takes place before the start of the war (e.g. the increase in the number of Ukrainian and English tweets). However, we do/did not know in advance how the treatment effect (the effect of the war) would evolve over time. Additionally, control units are not available as every user is "treated" with the war. Hence, the RDiT assumptions are violated without the possibility for a correction. Apart from this, by using a GAMM, as we do for the main results of our paper, we allow for a more flexible non-linear fit, which would not be possible with a RDIT without a polynomial fit (bias!). This in in turn allows us to capture subtleties in the behavioural changes of the users over time. Additionally, one can measure effect sizes between any two points in time with our chosen approach.

Nonetheless, as a robustness check, we construct the following RDiT design, in which we assume a time-constant effect. As observed in the behavioural effect plots of the GAMM, we see an immediate effect with the outbreak of the war (e.g. increase in number of tweets across all languages) and a short- to medium-term change in trend after (e.g. decrease in English and Russian number of tweets). Hence, we choose a window size of 16 weeks ($\sim$4 months) before and 16 weeks after the war, in which we look at changes in behaviour. More specifically, to model the number of tweets $Y_{t,u,l}$, which follow a Poisson distribution with intensity $\lambda_{t,u,l}$, we set

$$\lambda_{t,u,l} = exp(\mu_0 + \mu_{war,l}\mathbb{1}_{\{t>15\}} + \beta_{0,l}t + \beta_{war,l}\mathbb{1}_{\{t>15\}}(t-16) + W_{u,l}),$$

with $t = \{0, 1, ...15, 17, 18, ..., 32\}$ and we discard week $t = 16$, in which the war breaks out to measure the effect more cleanly. As before, μ is a general time-constant intercept[1] and the $W_{u,l}$ are language-specific time-constant random intercepts. Instead of a smooth global time trend, we now assume a linear behavioural trend $\beta_{0,l}$ for each language. With the outbreak of the war, the tweeting behaviour can shift immediately by $\mu_{war,l}$ and the linear trend can change by $\beta_{war,l}$.

The regression table of the fitted model is provided in Table 2. A visualization of the fitted behavioural effects is shown in Figure 4. As we can observe, the outbreak of the war leads to highly significant changes both instantaneously as well as in the short- to medium-term trend across all languages. The patterns are very similar to those demonstrated with the GAMM. Already before the war, users start tweeting slightly more in EN (+1.25% per week), but with the outbreak there is a considerable surge (+56.88%). Afterwards the initial trend reverses and EN tweeting behaviour decreases again (-6.59% per week, calculated by summing up both EN trend coefficients in the $exp(.)$). For RU, before the war, there is a minor decrease over time (-1.07% per week). As the war breaks out, users are tweeting slightly more in RU (+8.55%), but subsequently the negative trend strengthens (-4.43% per week). Finally, for UA, we observe a small positive trend from the start (+0.58% per week) and a medium-sized jump with the war outbreak (+8.61%). The positive trend speeds up in the weeks after (+0.96% per week). Overall, these behavioural patterns are nearly identical, or at least very similar, to the ones observed in the main paper using the GAMM.

[1] We could also introduce language-specific intercepts instead. However, the effects are picked up by the random user intercepts anyway and this way we can keep the specification as close as possible to the original one in the main paper.

Supplementary Table 2: Regression Discontinuity in Time - Tweet Model

	Dependent variable:
	Number of tweets
Trend - EN	0.0125***
	(0.0014)
Trend - RU	−0.0108***
	(0.0009)
Trend - UA	0.0058***
	(0.0007)
Intercept war - EN	0.4503***
	(0.0171)
Intercept war - RU	0.0821***
	(0.0123)
Intercept war - UA	0.0826***
	(0.0095)
Change in trend war - EN	−0.0807***
	(0.0018)
Change in trend war - RU	−0.0345***
	(0.0014)
Change in trend war - UA	0.0038***
	(0.0010)
Constant	−1.6354***
	(0.0209)
N	204,504
Expl. deviance	0.739

Notes: Poisson regression. The dependant variable is the number of tweets in the respective language, in the respective week for each active user. Random effects for all users are included. All coefficients are the linear predictors in the $exp(.)$. Values in parentheses the standard errors. *, ** and *** denote significance at the 10%, 5% and 1% level respectively.

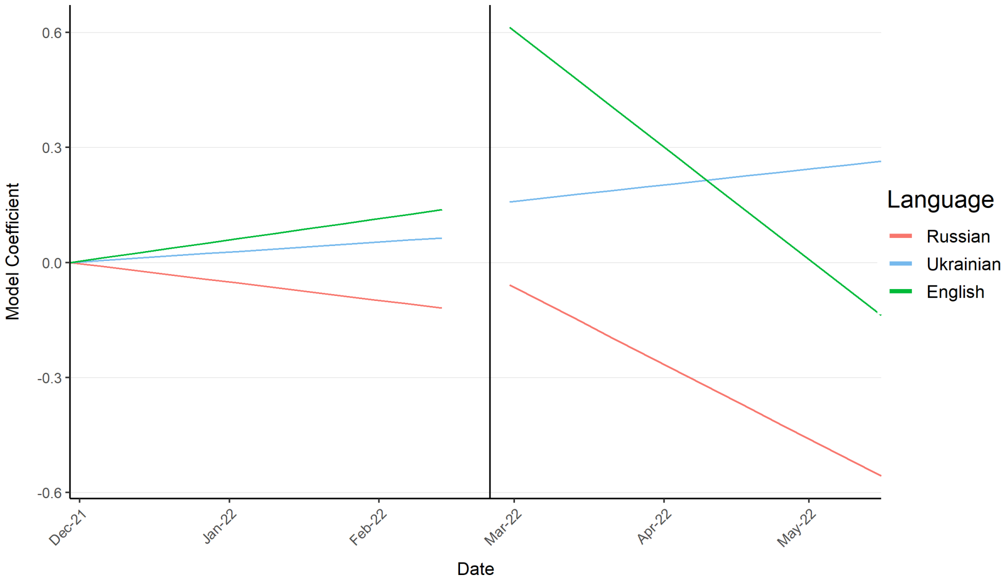

Supplementary Figure 4: Behavioural effects in the described RDiT design for the tweet model. The curves are constructed using the fitted trend coefficients and the intercept changes as listed in Table 2. We do not include the initial intercept. The vertical line denotes the outbreak of the war.

We repeat this RDiT strategy for the language models. Again we assume $X_{t,u} \sim Binomial(n_{t,u}, \pi_{t,u})$ and define the probability to tweet in one language over another as

$$\pi_{t,u} = f(\mu + \mu_{war,l}\mathbb{1}_{\{t>15\}} + \beta_0 t + \beta_{war,l}\mathbb{1}_{\{t>15\}}(t-16) + W_u(t)),$$

with $t = \{0, 1, ...15, 17, 18, ..., 32\}$, where $f(.)$ is the logistic function. The coefficients have the same interpretation as before (now with respect to the language probability). As in the main paper, we repeat this for all three language pairs. The resulting regression table is shown in Table 3. The fitted behavioural effects are illustrated in Figure 5. Similarly, we notice significant behavioural effects across the board with the outbreak of the war. For UA over RU, we observe a minor increase in the odds to tweet in UA before the war (+1.20% per week), a surge with the outbreak (+50.71%) and a much faster increase thereafter (+5.20% per week). Moving to UA over EN, the negative trend before the war is only weakly significant (-0.72% per week). With the war there is a major shift towards EN (-48.34% odds to tweet in UA), followed by a reversal of the trend (+8.59% per week). For RU over EN, the initial negative trend is non-significant (-0.19% per week). The outbreak of the war leads to a significant decrease in the odds (-51.14%), which is followed up by an upwards trend (+3.13%). In summary, the behavioural patterns for the choice of language closely mirror those demonstrated by the GAMM.

Supplementary Table 3: Regression Discontinuity in Time - Language Model

	Dependent variable:		
	Probability to tweet in		
	UA over RU	UA over EN	RU over EN
Trend	0.0119***	−0.0072*	−0.0019
	(0.0018)	(0.0042)	(0.0042)
Intercept war	0.4102***	−0.6606***	−0.7163***
	(0.0247)	(0.0503)	(0.0542)
Change in trend war	0.0388***	0.0896***	0.0327***
	(0.0026)	(0.0053)	(0.0056)
Constant	−0.4229***	0.9356***	1.2062***
	(0.0485)	(0.0889)	(0.0989)
Observations	34,697	32,733	31,144
Expl. deviance	0.844	0.928	0.913

Notes: Logistic regression. Dependant variable is the probability to tweet in one language over another in the respective week for each active user. Random effects for all users are included. All coefficients are the linear predictors in the logistic function. Values in parentheses the standard errors. *, ** and *** denote significance at the 10%, 5% and 1% level respectively.

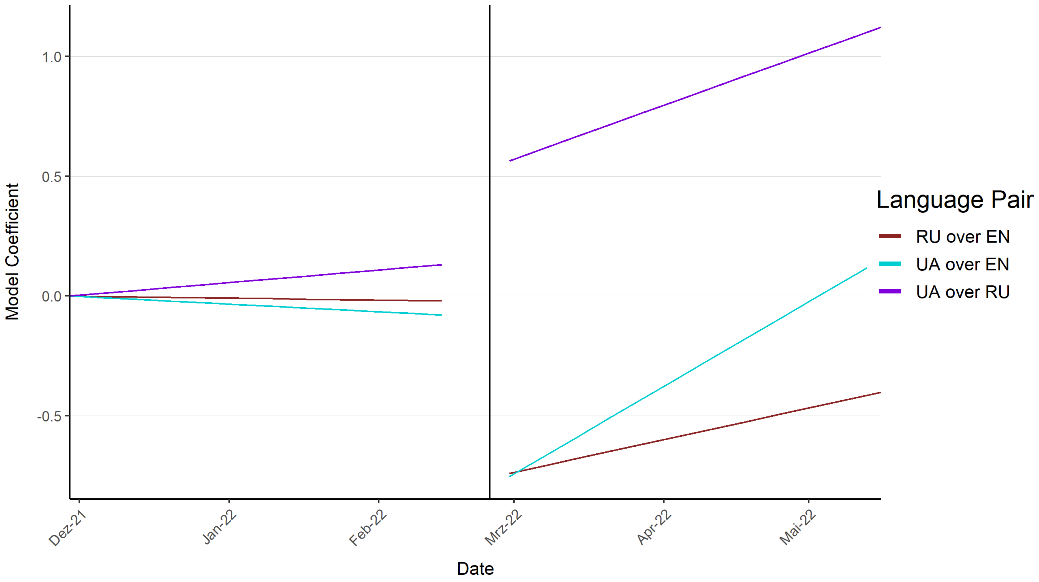

Supplementary Figure 5: Behavioural effects in the described RDiT design for the language model. The curves are constructed using the fitted trend coefficients and the intercept changes as listed in Table 3. We remove the initial intercepts from the plot to make comparison easier. The vertical line denotes the outbreak of the war.

Supplementary Notes 3: User Characteristics

Supplementary Table 4: Summary Statistics of User Characteristics

Characteristic	Mean	Min	25% Quantile	Median	75% Quantile	Max
Total # of tweets	4818.03	1.00	39.00	434.00	3348.25	394418.00
Followers	2156.46	0.00	4.00	41.00	234.00	8390808.00
Followings	332.14	0.00	24.00	84.00	266.00	114494.00
Likes	7167.39	0.00	39.00	510.00	4171.00	934293.00
Verified (0 or 1)	0.01	0.00	0.00	0.00	0.00	1.00
Account age (month)	70.61	0.01	20.81	62.11	120.57	633.39
Tweet frequency	7.90	0.00	0.16	0.69	2.77	4086.49
Likes frequency	10.70	0.00	0.10	0.74	4.33	9104.14
# of tweets in Ukraine	68.25	1.00	2.00	6.00	26.00	16209.00
War topic 1	6.00	0.00	0.00	0.00	2.00	1638.00
War topic 1 (rel.)	0.18	0.00	0.00	0.01	0.25	1.00
War topic 2	2.16	0.00	0.00	0.00	0.00	755.00
War topic 2 (rel.)	0.03	0.00	0.00	0.00	0.02	1.00

Notes: n = 41,696 users. All Twitter user attributes are a snapshot from the last time we observe a user's respective tweet in our sample. Total # of tweets reports the number of the number of tweets a user has tweeted in total (including outside the Ukraine). Followers are the number of accounts that follow a user. Followings reports the number of accounts a user is following. Likes the number of tweets a user has liked. Verified indicates if a user has a verified account (1) or not (0). The account age reports the number of months a user account has existed from account creation to their latest tweet in our dataset. The tweet frequency the number of tweets per day. The likes frequency the number of liked tweets (by the user) per day. # of Tweets in Ukraine reports the total number of tweets in our dataset. War topic 1 reports the number of tweets assigned to first war topic cluster (topic #1), which covers updates about the war and calls for help. War topic 1 (rel.) the relative share of tweets assigned to this topic. War topic 2 reports the number of tweets assigned to second war topic cluster (topic #3), which covers a more political side of the overall conflict. War topic 2 (rel.) the relative share. More information on the topics can be found in Supplementary Notes 1:.

Supplementary Notes 4: Language Distribution

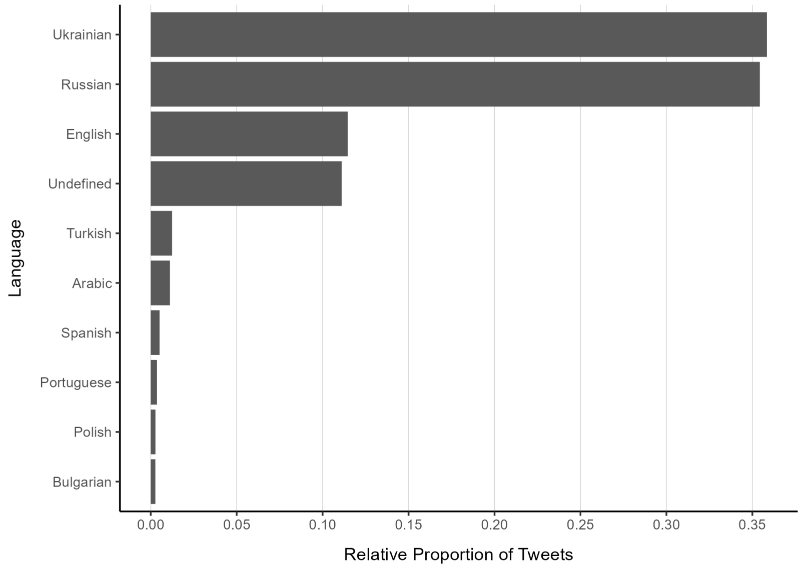

Supplementary Figure 6: Relative distribution of the top 10 languages across the entire sample after preprocessing and cleaning (n = 2,845,670 tweets). "Undefined" consists of tweets that are too short, contain only hashtags, contain only mentions or only have media (links), for all of which a language is not available.

Supplementary Notes 5: Complete User Activity Graphs

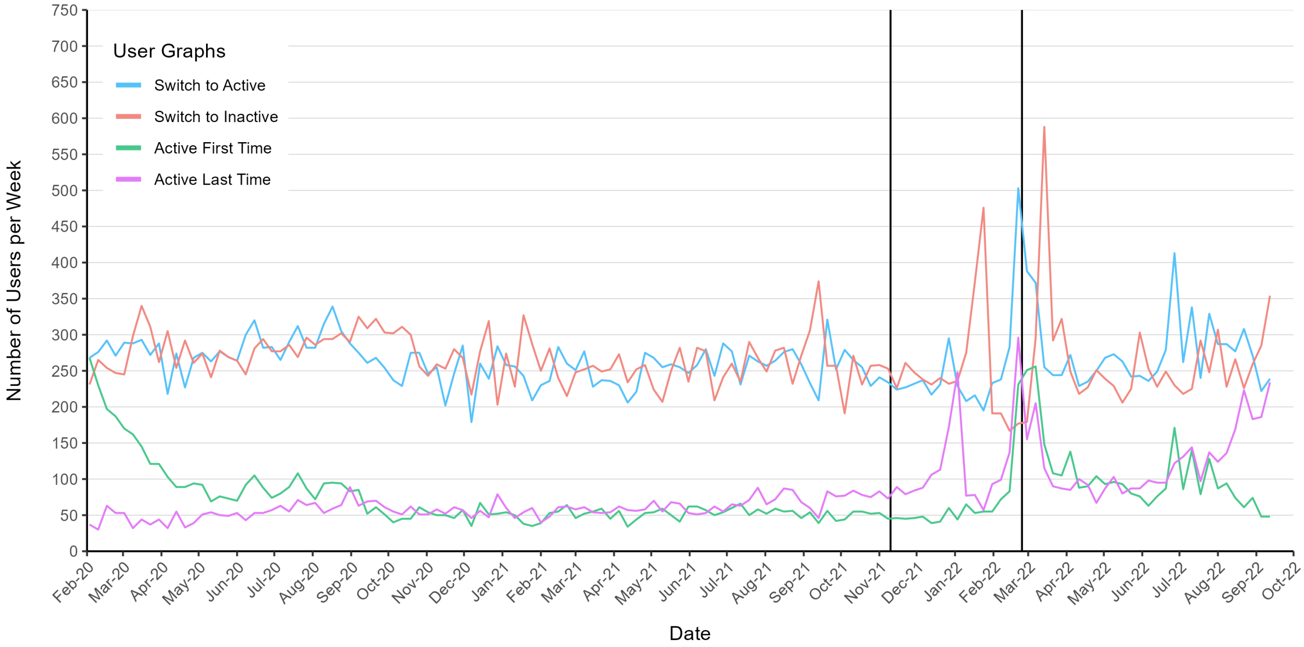

Supplementary Figure 7: Weekly user activity graphs without the number of active users. The figure corresponds to Figure 2 in the main text after removing the active user graph and rescaling the y-axis. The blue (red) graph reports the number of users who switch to active (inactive), the green the number of users who switch to active for the first time, the purple the number of users who were active for the last time, i.e. drop out of the sample altogether. The graphs are skewed upwards respectively downwards towards beginning and end of the analysis period due to the nature of how the dataset is constructed. The first vertical line denotes the mobilization of the Russian troops along the Ukrainian border. The second line denotes the outbreak of the war.

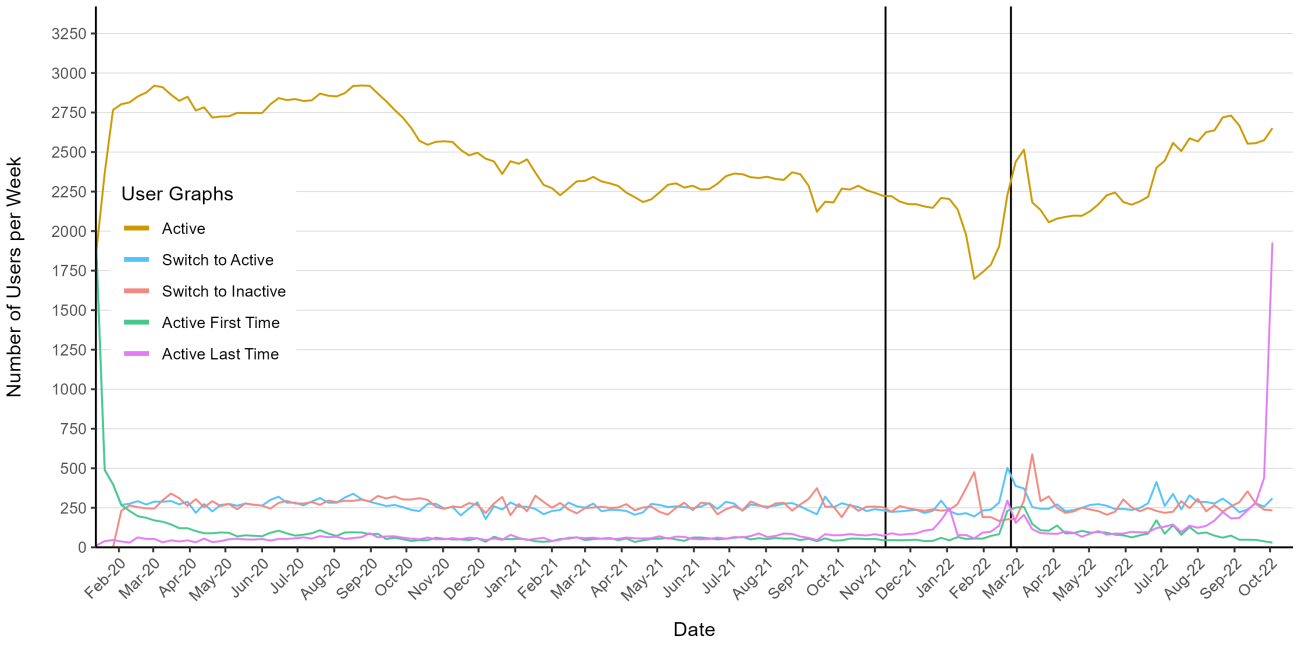

Supplementary Figure 8: Complete weekly user activity graphs. The figure corresponds to Figure2 in the main text without the filtering of the first and last three weeks of the analysis period. The brown graph reports the number of active users in each week. The blue (red) graph reports the number of users who switch to active (inactive), the green the number of users who switch to active for the first time, the purple the number of users who were active for the last time, i.e. drop out of the sample altogether. The graphs are skewed upwards respectively downwards towards beginning and end of the analysis period due to the nature of how the dataset is constructed. The first vertical line denotes the mobilization of the Russian troops along the Ukrainian border. The second line denotes the outbreak of the war.

Supplementary Notes 6: Four-weekly Sample & Behavioural Changes

Figure 9 and Figure 10 visualize the four-weekly changes for both tweeting activity and language choice. In the plots, each rectangle captures the sample respectively behavioural shift from the first day of the given four week period, to the first day of the next four week period. The numbers in the rectangles note the exact change in %. Blue colors indicate an increase, red a decrease. The first vertical line denotes the mobilization of the Russian troops along the Ukrainian border. The second line denotes the outbreak of the war. As evident from the plots (darker colours), the largest shifts, both behavioural and sample, take place shortly before, with and after the outbreak of the war.

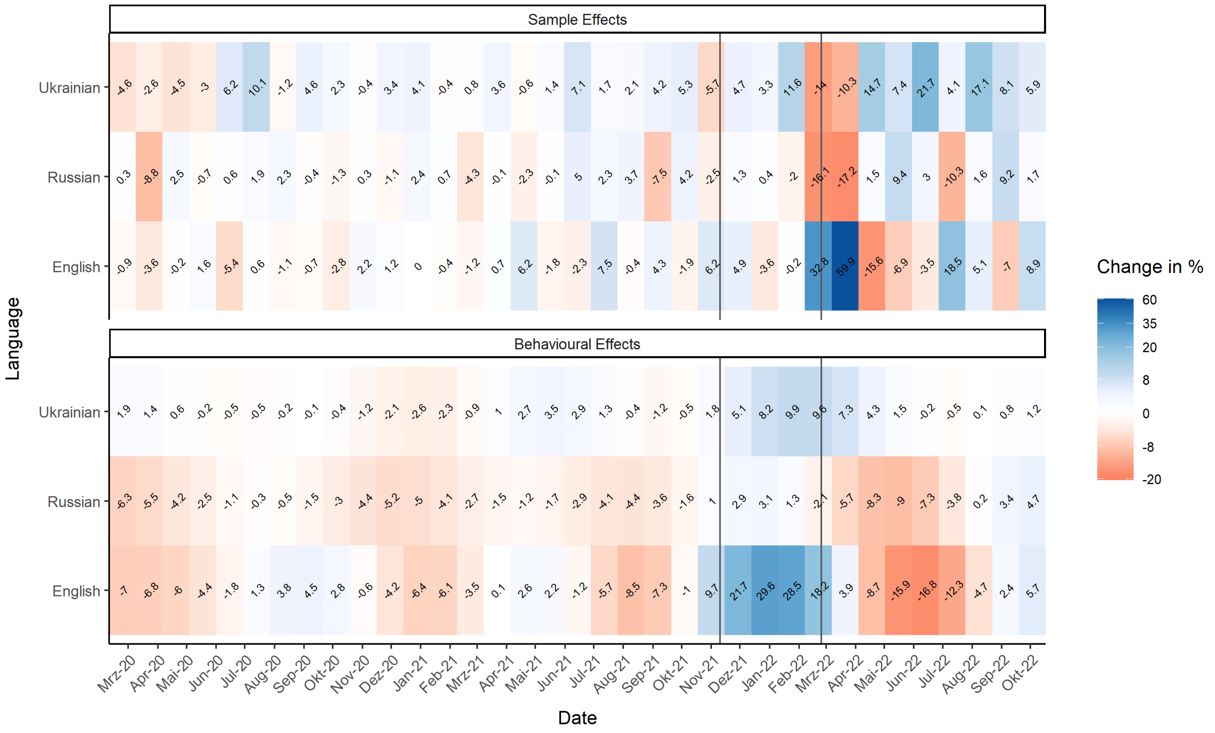

Supplementary Figure 9: Four-weekly sample and behavioural changes for tweeting activity.

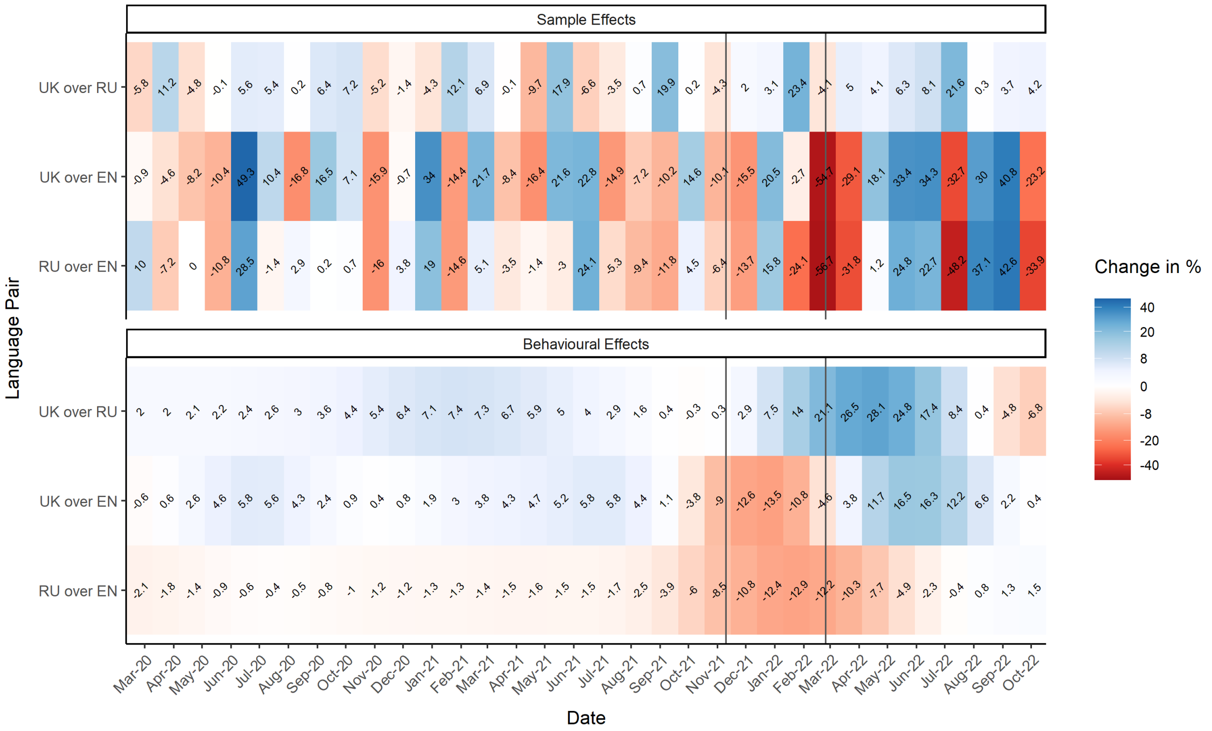

Supplementary Figure 10: Four-weekly sample and behavioural changes for language choice.

Supplementary Notes 7: Random Effect Distribution (Tweet Model) over Time

The figures reported here provide an in-depth look into the distribution of the random effects of the tweet model over time. For this, we form groups of six consecutive weeks. With 143 analysis weeks in total, this gives us a total of 24 groups, with the last group only consisting of five weeks (the last five of the analysis period). We then create density-based violin plots for the distribution of the random effects for each of the six-weekly groups, for each of the three languages separately, and report them (over time) in the figures below. The figures allow a closer look how the distribution of the random effects and thus the sample changes over time. At the upper tail, we have users who tweet a lot in the respective language on average, at the lower tail who do (almost) not tweet at all.

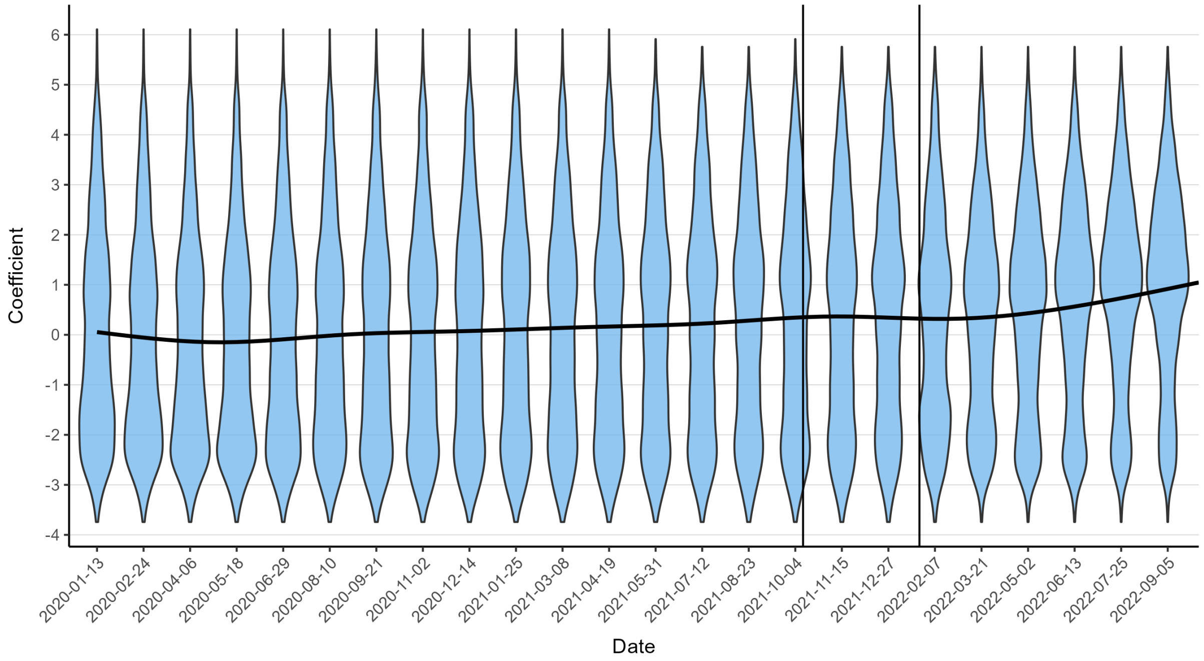

Supplementary Figure 11: Ukrainian Violin Plots. Each density-based violin plot is thicker (thinner) in the respective area, if there are more (less) users active in the given six-weekly group (x-axis) with the respective fitted UA random effect coefficient (y-axis). The black horizontal line denotes the smoothed average as reported in Figure 3b. The first vertical line denotes the mobilization of the Russian troops along the Ukrainian border. The second vertical line denotes the outbreak of the war.

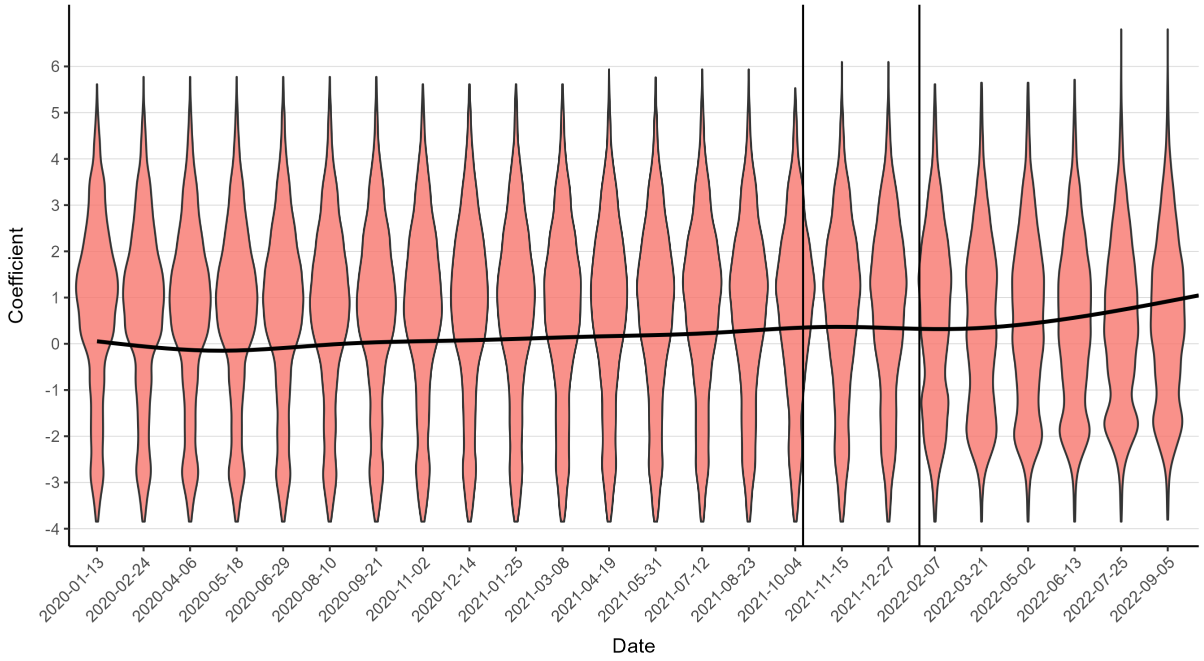

Supplementary Figure 12: Russian Violin Plots. Each density-based violin plot is thicker (thinner) in the respective area, if there are more (less) users active in the given six-weekly group (x-axis) with the respective fitted RU random effect coefficient (y-axis). The black horizontal line denotes the smoothed average as reported in Figure 3b. The first vertical line denotes the mobilization of the Russian troops along the Ukrainian border. The second vertical line denotes the outbreak of the war.

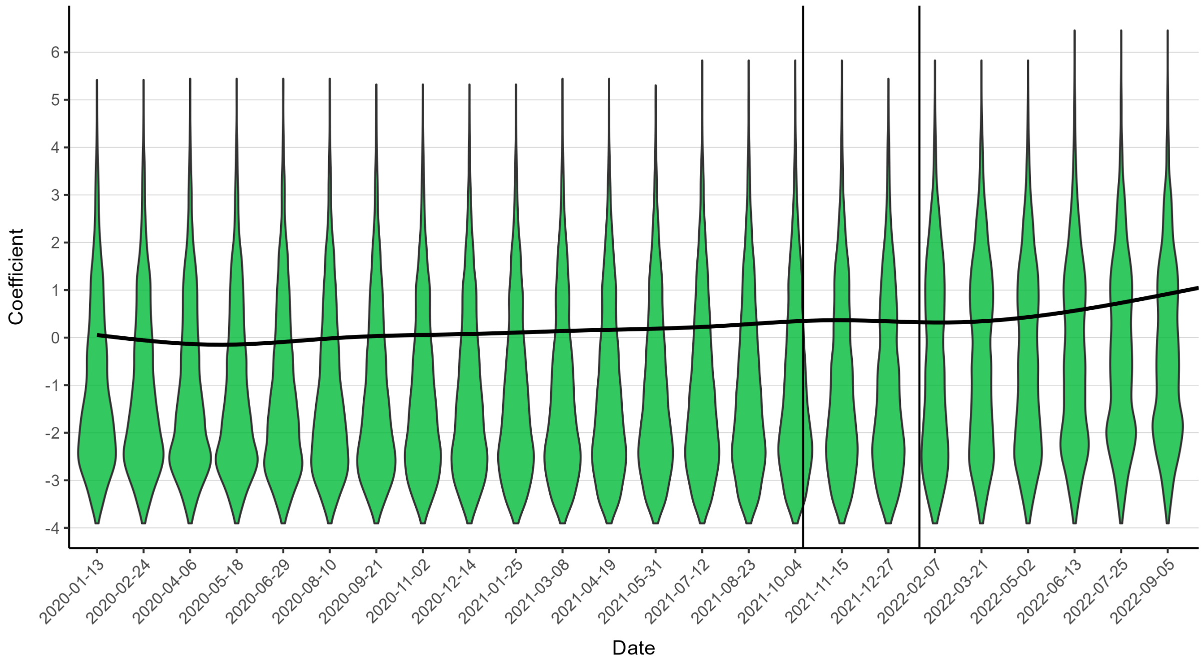

Supplementary Figure 13: English Violin Plots. Each density-based violin plot is thicker (thinner) in the respective area, if there are more (less) users active in the given six-weekly group (x-axis) with the respective fitted EN random effect coefficient (y-axis). The black horizontal line denotes the smoothed average as reported in Figure 3b. The first vertical line denotes the mobilization of the Russian troops along the Ukrainian border. The second vertical line denotes the outbreak of the war.

Supplementary Notes 8: Random Effect Distribution (Language Models) over Time

The figures reported here provide an in-depth look into the distribution of the random effects of the language models over time. We follow the same strategy as we employ for the tweet model, described in supplementary material Supplementary Notes 7:.

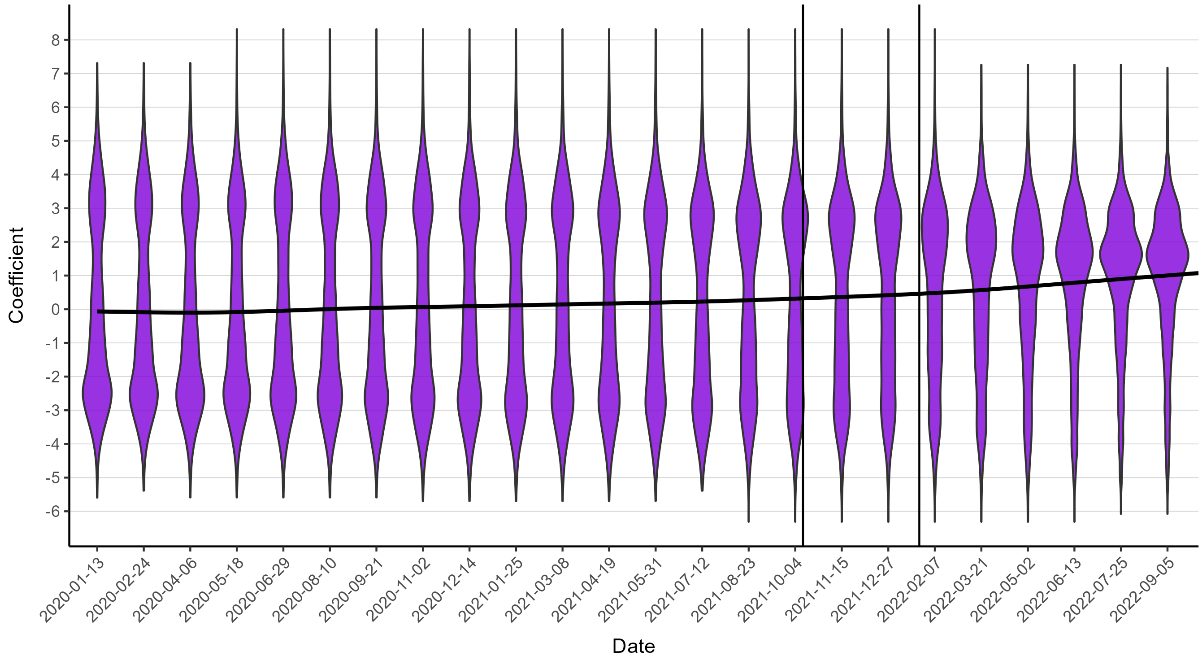

Supplementary Figure 14: UA over RU Violin Plots. Each density-based violin plot is thicker (thinner) in the respective area, if there are more (less) users active in the given six-weekly group (x-axis) with the respective fitted random effect coefficient (y-axis) of the UA over RU language model. The black horizontal line denotes the smoothed average as reported in Figure 4b. The first vertical line denotes the mobilization of the Russian troops along the Ukrainian border. The second vertical line denotes the outbreak of the war.

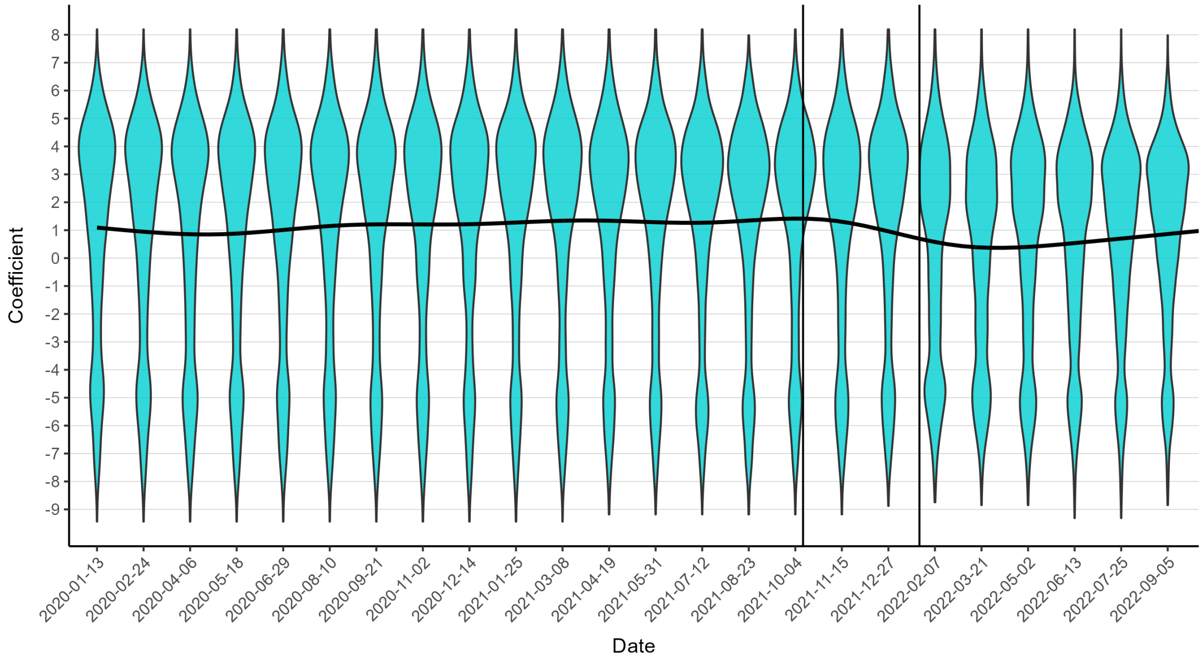

Supplementary Figure 15: UA over EN Violin Plots. Each density-based violin plot is thicker (thinner) in the respective area, if there are more (less) users active in the given six-weekly group (x-axis) with the respective fitted RU random effect coefficient (y-axis) of the UA over EN language model. The black horizontal line denotes the smoothed average as reported in Figure 4b. The first vertical line denotes the mobilization of the Russian troops along the Ukrainian border. The second vertical line denotes the outbreak of the war.

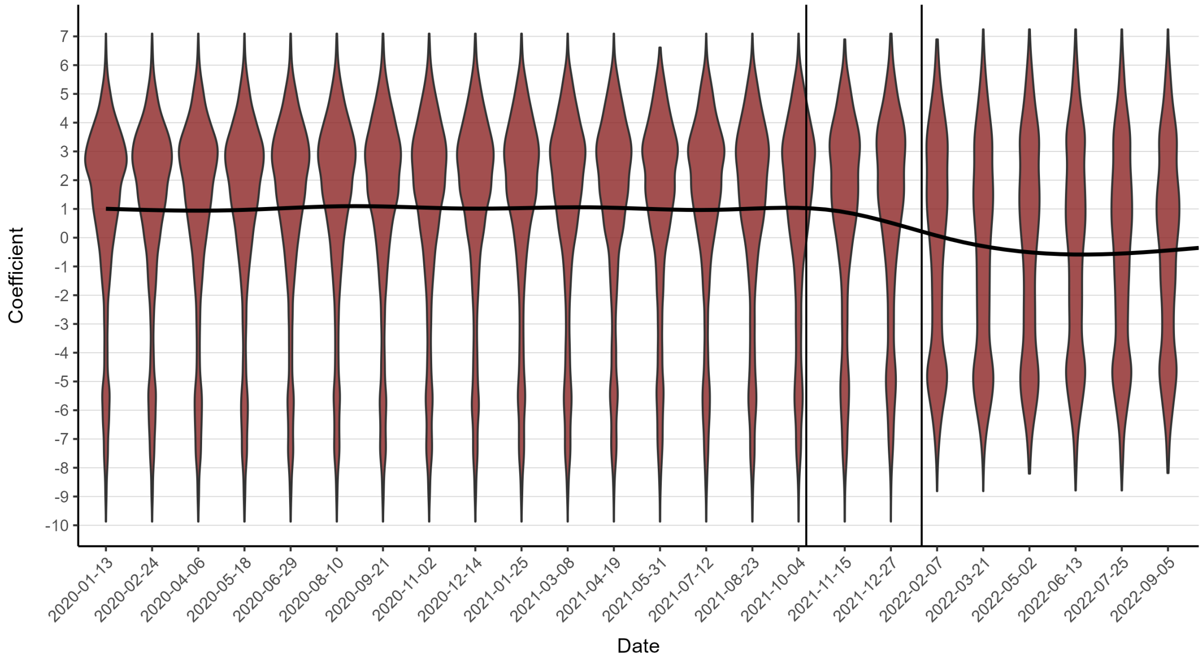

Supplementary Figure 16: RU over EN Violin Plots. Each density-based violin plot is thicker (thinner) in the respective area, if there are more (less) users active in the given six-weekly group (x-axis) with the respective fitted EN random effect coefficient (y-axis) of the RU over EN language model.. The black horizontal line denotes the smoothed average as reported in Figure 4b. The first vertical line denotes the mobilization of the Russian troops along the Ukrainian border. The second vertical line denotes the outbreak of the war.

Supplementary Notes 9: Weekly Changes (Speed) for Sample and Behavioural Effects

The tables provided in this section are an alternative to Table 1 and Table 2 in the main paper. Here, we measure the speed of change (instead of the total change) between the key dates in our study period. We calculate this, by measuring the total change and dividing it by the length (number of weeks) of the interval, which gives us an average weekly change. Note, that this smooths out any sudden changes happening between the two respective dates. If one is interested in weekly or four-weekly changes, we refer to Figure 3 and Figure 4 as well as respectively Figure 7 and Figure 8 in the main paper. Manual calculations between any two weeks of the study period are also possible using the coefficient tables Table 7 and Table 8.

Supplementary Table 5: Tweet Activity Weekly Change (Speed) between Key Dates

Language	Sample Effects			
	Start - Aggression	Aggression - War	War - End Study	Aggression - End Study
English	+0.066%	+2.325%	+1.661%	+2.267%
Ukrainian	+0.463%	-0.029%	+2.741%	+1.848%
Russian	-0.0314%	-1.161%	-0.129%	-0.443%
	Behavioural Effects			
English	-0.370%	+8.674%	-1.249%	+0.810%
Ukrainian	+0.050%	+2.381%	+0.475%	+1.198%
Russian	-0.526%	+0.312%	-0.746%	-0.432%

Notes: Speed of change of tweeting activity both sample and behavioural effects between key dates. For the start date calculation, we drop the first two weeks of the study period. Start: start of the study period—27th January 2020. Aggression: first official US report of a mobilization of the Russian troops along the Ukrainian border—11th November 2021. War: outbreak of the war—24th February 2022. End Study: end of the study period—10th October 2022.

Supplementary Table 6: Language Choice Weekly Change (Speed) between Key Dates

Language	Sample Effects			
	Start - Aggression	Aggression - War	War - End Study	Aggression - End Study
UA over RU	+0.711%	+0.866%	+2.054%	+1.856%
UA over EN	+0.230%	-3.472%	+1.311%	-0.6804%
RU over EN	-0.204%	-4.116%	-0.9166%	-1.552%
	Behavioural Effects			
UA over RU	+1.384%	+3.472%	+4.039%	+5.290%
UA over EN	+0.690%	-2.241%	+2.896%	+0.594%
RU over EN	-0.411%	-2.580%	-0.646%	-1.093%

Notes: Speed of change of language choice for both sample and behavioural effects between key dates. For the start date calculation, we drop the first two weeks of the study period. Start: start of the study period—27th January 2020. Aggression: first official US report of a mobilization of the Russian troops along the Ukrainian border—11th November 2021. War: outbreak of the war—24th February 2022. End Study: end of the study period—10th October 2022.

Supplementary Notes 10: Model Coefficients

Supplementary Table 7: Weekly Model Coefficients - Tweet Model

Date	Sample Coefficients			Behavioural Coefficients		
	UA	RU	EN	UA	RU	EN
2020-01-13	0.1444	0.9239	-1.0735	-0.1385	0.5409	0.1984
2020-01-20	0.0474	0.7898	-1.0984	-0.1335	0.5242	0.1802
2020-01-27	-0.0206	0.7386	-1.0885	-0.1286	0.5075	0.1620
2020-02-03	-0.0221	0.7424	-1.0809	-0.1237	0.4909	0.1438
2020-02-10	-0.0349	0.7283	-1.0704	-0.1190	0.4745	0.1257
2020-02-17	-0.0555	0.7582	-1.0938	-0.1144	0.4583	0.1075
2020-02-24	-0.0681	0.7419	-1.0980	-0.1101	0.4426	0.0895
2020-03-02	-0.0849	0.7391	-1.1166	-0.1061	0.4274	0.0717
2020-03-09	-0.0737	0.6995	-1.1204	-0.1025	0.4128	0.0540
2020-03-16	-0.0689	0.6772	-1.1144	-0.0993	0.3989	0.0366
2020-03-23	-0.0947	0.6494	-1.1342	-0.0966	0.3859	0.0196
2020-03-30	-0.1231	0.6638	-1.1280	-0.0944	0.3737	0.0030
2020-04-06	-0.1162	0.6839	-1.1418	-0.0927	0.3626	-0.0129
2020-04-13	-0.1447	0.6676	-1.1346	-0.0916	0.3525	-0.0281
2020-04-20	-0.1411	0.6743	-1.1366	-0.0909	0.3434	-0.0423
2020-04-27	-0.1573	0.6701	-1.1489	-0.0908	0.3355	-0.0555
2020-05-04	-0.1500	0.6957	-1.1309	-0.0910	0.3286	-0.0674
2020-05-11	-0.1693	0.6612	-1.1208	-0.0917	0.3228	-0.0779
2020-05-18	-0.1713	0.6676	-1.1203	-0.0926	0.3180	-0.0869
2020-05-25	-0.1843	0.6640	-1.1309	-0.0938	0.3142	-0.0941
2020-06-01	-0.1759	0.6765	-1.1395	-0.0952	0.3111	-0.0995
2020-06-08	-0.1400	0.6677	-1.1564	-0.0966	0.3089	-0.1031
2020-06-15	-0.1115	0.6739	-1.1757	-0.0981	0.3072	-0.1046
2020-06-22	-0.0915	0.6820	-1.2066	-0.0995	0.3060	-0.1042
2020-06-29	-0.0772	0.6805	-1.2027	-0.1008	0.3052	-0.1018
2020-07-06	-0.0432	0.6910	-1.1907	-0.1019	0.3046	-0.0975
2020-07-13	-0.0154	0.6922	-1.1697	-0.1029	0.3040	-0.0915
2020-07-20	-0.0265	0.6916	-1.1500	-0.1036	0.3032	-0.0839
2020-07-27	-0.0267	0.6952	-1.1540	-0.1041	0.3023	-0.0749
2020-08-03	-0.0212	0.7165	-1.1545	-0.1045	0.3009	-0.0649
2020-08-10	-0.0272	0.7148	-1.1805	-0.1047	0.2989	-0.0541
2020-08-17	-0.0074	0.7051	-1.1905	-0.1048	0.2963	-0.0429
2020-08-24	0.0078	0.7167	-1.1934	-0.1048	0.2929	-0.0315
2020-08-31	0.0195	0.7350	-1.1914	-0.1050	0.2886	-0.0205
2020-09-07	0.0176	0.7112	-1.1876	-0.1052	0.2834	-0.0101
2020-09-14	0.0119	0.7099	-1.1817	-0.1056	0.2772	-0.0008
2020-09-21	0.0034	0.6975	-1.1891	-0.1064	0.2700	0.0072
2020-09-28	0.0250	0.6976	-1.2144	-0.1075	0.2618	0.0135
2020-10-05	0.0401	0.6983	-1.2164	-0.1092	0.2527	0.0178
2020-10-12	0.0824	0.6656	-1.2213	-0.1113	0.2426	0.0199
2020-10-19	0.0638	0.6919	-1.1966	-0.1140	0.2317	0.0196
2020-10-26	0.0406	0.6791	-1.1877	-0.1174	0.2200	0.0170
2020-11-02	0.0364	0.7012	-1.1948	-0.1214	0.2076	0.0118
2020-11-09	0.0114	0.6888	-1.1880	-0.1260	0.1948	0.0043
2020-11-16	0.0364	0.6961	-1.1595	-0.1312	0.1816	-0.0055
2020-11-23	0.0645	0.6971	-1.1592	-0.1369	0.1682	-0.0174
2020-11-30	0.0702	0.6902	-1.1832	-0.1431	0.1547	-0.0311
2020-12-07	0.0892	0.7030	-1.1895	-0.1496	0.1413	-0.0463
2020-12-14	0.0818	0.7181	-1.1792	-0.1563	0.1282	-0.0626
2020-12-21	0.1195	0.7466	-1.1615	-0.1631	0.1154	-0.0796
2020-12-28	0.1106	0.7134	-1.1834	-0.1699	0.1032	-0.0969
2021-01-04	0.1026	0.7416	-1.1908	-0.1764	0.0917	-0.1139
2021-01-11	0.0805	0.7212	-1.1848	-0.1825	0.0809	-0.1303
2021-01-18	0.0802	0.7370	-1.1908	-0.1880	0.0709	-0.1457
2021-01-25	0.1069	0.7200	-1.1871	-0.1928	0.0618	-0.1596
2021-02-01	0.1210	0.6864	-1.1911	-0.1967	0.0537	-0.1717
2021-02-08	0.1275	0.6738	-1.1845	-0.1996	0.0464	-0.1818
2021-02-15	0.1088	0.6916	-1.1885	-0.2014	0.0401	-0.1896
2021-02-22	0.1146	0.6759	-1.1994	-0.2021	0.0345	-0.1951
2021-03-01	0.1380	0.6961	-1.2095	-0.2014	0.0298	-0.1981
2021-03-08	0.1606	0.6854	-1.2033	-0.1996	0.0257	-0.1989
2021-03-15	0.1469	0.6979	-1.1933	-0.1965	0.0221	-0.1974
2021-03-22	0.1495	0.6750	-1.1923	-0.1922	0.0190	-0.1940
2021-03-29	0.1677	0.6682	-1.2176	-0.1868	0.0161	-0.1890
2021-04-05	0.2003	0.6532	-1.1828	-0.1804	0.0132	-0.1827
2021-04-12	0.1633	0.6451	-1.1456	-0.1732	0.0104	-0.1757
2021-04-19	0.1430	0.6515	-1.1321	-0.1653	0.0073	-0.1685
2021-04-26	0.1316	0.6788	-1.1236	-0.1569	0.0038	-0.1614
2021-05-03	0.1181	0.6996	-1.1593	-0.1482	-0.0002	-0.1552
2021-05-10	0.1485	0.6813	-1.1557	-0.1395	-0.0049	-0.1502
2021-05-17	0.1567	0.6503	-1.1499	-0.1309	-0.0102	-0.1470
2021-05-24	0.2021	0.6460	-1.1536	-0.1227	-0.0164	-0.1460
2021-05-31	0.1768	0.6284	-1.1466	-0.1150	-0.0234	-0.1475
2021-06-07	0.2050	0.6547	-1.1489	-0.1081	-0.0313	-0.1519
2021-06-14	0.2252	0.6988	-1.1732	-0.1020	-0.0400	-0.1593
2021-06-21	0.2084	0.6901	-1.1534	-0.0970	-0.0495	-0.1697
2021-06-28	0.2151	0.6460	-1.1293	-0.0931	-0.0597	-0.1831
2021-07-05	0.2331	0.6823	-1.1127	-0.0903	-0.0705	-0.1992
2021-07-12	0.2417	0.7217	-1.1012	-0.0886	-0.0817	-0.2179
2021-07-19	0.2429	0.7644	-1.1254	-0.0881	-0.0932	-0.2385
2021-07-26	0.2615	0.7729	-1.1267	-0.0887	-0.1048	-0.2606
2021-08-02	0.2809	0.7699	-1.1248	-0.0901	-0.1162	-0.2835
2021-08-09	0.2621	0.7585	-1.1055	-0.0923	-0.1273	-0.3065
2021-08-16	0.2567	0.7780	-1.1075	-0.0951	-0.1377	-0.3287
2021-08-23	0.2904	0.7340	-1.0934	-0.0983	-0.1475	-0.3493
2021-08-30	0.2863	0.6938	-1.0905	-0.1015	-0.1562	-0.3675

2021-09-06	0.3032	0.6806	-1.0638	-0.1046	-0.1637	-0.3823
2021-09-13	0.2873	0.6779	-1.1090	-0.1073	-0.1699	-0.3929
2021-09-20	0.3367	0.7338	-1.0799	-0.1092	-0.1747	-0.3986
2021-09-27	0.3577	0.7364	-1.0825	-0.1102	-0.1778	-0.3987
2021-10-04	0.3551	0.7216	-1.0832	-0.1099	-0.1794	-0.3926
2021-10-11	0.3244	0.7182	-1.0722	-0.1081	-0.1793	-0.3799
2021-10-18	0.2989	0.7106	-1.0661	-0.1047	-0.1776	-0.3603
2021-10-25	0.3072	0.6990	-1.0363	-0.0994	-0.1744	-0.3337
2021-11-01	0.2960	0.6966	-1.0230	-0.0922	-0.1698	-0.3001
2021-11-08	0.3379	0.7090	-1.0287	-0.0829	-0.1640	-0.2597
2021-11-15	0.3372	0.6735	-0.9939	-0.0715	-0.1572	-0.2131
2021-11-22	0.3448	0.6882	-0.9587	-0.0580	-0.1496	-0.1607
2021-11-29	0.3418	0.7095	-0.9749	-0.0426	-0.1415	-0.1034
2021-12-06	0.3718	0.7259	-0.9798	-0.0252	-0.1332	-0.0420
2021-12-13	0.3615	0.7073	-0.9739	-0.0061	-0.1251	0.0224
2021-12-20	0.3694	0.7168	-0.9699	0.0145	-0.1174	0.0888
2021-12-27	0.3745	0.7140	-1.0113	0.0364	-0.1106	0.1559
2022-01-03	0.3903	0.7400	-1.0135	0.0593	-0.1049	0.2224
2022-01-10	0.4119	0.7564	-1.0142	0.0830	-0.1006	0.2871
2022-01-17	0.4725	0.7771	-0.9818	0.1070	-0.0981	0.3488
2022-01-24	0.4843	0.6937	-1.0129	0.1312	-0.0975	0.4063
2022-01-31	0.4441	0.6866	-1.0103	0.1551	-0.0991	0.4584
2022-02-07	0.4352	0.6483	-0.9339	0.1785	-0.1030	0.5042
2022-02-14	0.3877	0.6269	-0.8851	0.2011	-0.1094	0.5429
2022-02-21	0.3335	0.5177	-0.7295	0.2225	-0.1182	0.5737
2022-02-28	0.2587	0.4196	-0.5502	0.2427	-0.1295	0.5962
2022-03-07	0.2151	0.3416	-0.3504	0.2613	-0.1432	0.6100
2022-03-14	0.1871	0.3176	-0.2198	0.2782	-0.1592	0.6151
2022-03-21	0.2244	0.3285	-0.2601	0.2933	-0.1772	0.6117
2022-03-28	0.3218	0.3511	-0.3764	0.3066	-0.1971	0.5999
2022-04-04	0.3584	0.3292	-0.3820	0.3179	-0.2184	0.5803
2022-04-11	0.3517	0.3162	-0.4079	0.3274	-0.2410	0.5537
2022-04-18	0.3615	0.3430	-0.4296	0.3350	-0.2644	0.5208
2022-04-25	0.3664	0.3740	-0.4730	0.3409	-0.2882	0.4826
2022-05-02	0.3568	0.4058	-0.4820	0.3452	-0.3120	0.4402
2022-05-09	0.3792	0.4161	-0.4845	0.3481	-0.3355	0.3948
2022-05-16	0.4333	0.4330	-0.5006	0.3498	-0.3582	0.3475
2022-05-23	0.4643	0.4319	-0.5049	0.3504	-0.3796	0.2996
2022-05-30	0.5723	0.4557	-0.5206	0.3502	-0.3996	0.2522
2022-06-06	0.6094	0.4583	-0.5272	0.3494	-0.4177	0.2064
2022-06-13	0.6297	0.4630	-0.5362	0.3482	-0.4336	0.1632
2022-06-20	0.6475	0.4414	-0.4730	0.3469	-0.4472	0.1235
2022-06-27	0.6184	0.3951	-0.4034	0.3455	-0.4583	0.0881
2022-07-04	0.6542	0.3870	-0.3916	0.3442	-0.4668	0.0575
2022-07-11	0.6700	0.3548	-0.3663	0.3433	-0.4725	0.0321
2022-07-18	0.7292	0.3758	-0.3592	0.3427	-0.4757	0.0121
2022-07-25	0.7320	0.3269	-0.2908	0.3426	-0.4762	-0.0023
2022-08-01	0.7838	0.3654	-0.3129	0.3430	-0.4744	-0.0115
2022-08-08	0.8281	0.3705	-0.3162	0.3438	-0.4703	-0.0156
2022-08-15	0.8730	0.3804	-0.3474	0.3452	-0.4643	-0.0152
2022-08-22	0.8538	0.3812	-0.3686	0.3471	-0.4566	-0.0107
2022-08-29	0.8797	0.4186	-0.3724	0.3493	-0.4475	-0.0029
2022-09-05	0.9061	0.4585	-0.3889	0.3519	-0.4372	0.0076
2022-09-12	0.9683	0.4848	-0.4006	0.3547	-0.4262	0.0201
2022-09-19	0.9985	0.5119	-0.3948	0.3577	-0.4147	0.0338
2022-09-26	1.0007	0.4998	-0.3553	0.3608	-0.4029	0.0483
2022-10-03	0.9631	0.4756	-0.3033	0.3639	-0.3910	0.0631

Notes: Coefficients for both sample and behavioural effects in the tweet model for each week. The sample coefficients are the averages of the random effects of the active users in each week. The behavioural coefficients are directly extracted from the global smooth curve fitted in the GAMM. Shifts (effect sizes) between any two weeks can be calculated through $exp(coef_{week2} - coef_{week1})$ and are % changes in tweeting activity between those two weeks.

Supplementary Table 8: Weekly Model Coefficients - Language Models

Date	Sample Coefficients			Behavioural Coefficients		
	UA over RU	UA over EN	RU over EN	UA over RU	UA over EN	RU over EN
2020-01-13	-0.0419	1.0399	1.0090	-0.8256	-0.3333	0.4483
2020-01-20	-0.0047	1.0484	1.0098	-0.8206	-0.3355	0.4429
2020-01-27	-0.1069	0.9828	0.9318	-0.8156	-0.3377	0.4375
2020-02-03	-0.0994	1.0424	1.0436	-0.8106	-0.3397	0.4321
2020-02-10	-0.0606	0.9146	0.8597	-0.8056	-0.3415	0.4268
2020-02-17	-0.0961	1.0858	1.0324	-0.8006	-0.3428	0.4216
2020-02-24	-0.1664	0.9734	1.0274	-0.7956	-0.3435	0.4165
2020-03-02	-0.0487	1.1451	1.0453	-0.7906	-0.3435	0.4116
2020-03-09	0.0632	0.9463	0.8698	-0.7856	-0.3426	0.4069
2020-03-16	-0.0635	0.9165	0.9289	-0.7805	-0.3407	0.4024
2020-03-23	-0.0602	0.9258	0.9531	-0.7754	-0.3376	0.3981
2020-03-30	-0.1376	0.7700	0.8945	-0.7703	-0.3333	0.3942
2020-04-06	-0.1077	0.7997	0.9657	-0.7651	-0.3276	0.3905
2020-04-13	-0.1413	0.8706	0.9638	-0.7599	-0.3207	0.3872
2020-04-20	-0.1093	0.8405	0.9527	-0.7546	-0.3124	0.3842
2020-04-27	-0.1716	0.7230	0.8845	-0.7492	-0.3028	0.3815
2020-05-04	-0.1805	0.8298	0.9737	-0.7438	-0.2921	0.3790
2020-05-11	-0.1218	0.6580	0.8818	-0.7382	-0.2803	0.3769
2020-05-18	-0.1101	0.7303	0.8383	-0.7326	-0.2676	0.3750
2020-05-25	-0.2037	0.7411	0.8881	-0.7268	-0.2541	0.3733
2020-06-01	-0.0763	0.9203	0.9822	-0.7210	-0.2401	0.3718
2020-06-08	-0.0681	1.0685	1.0648	-0.7150	-0.2258	0.3705
2020-06-15	-0.0552	1.1309	1.0887	-0.7089	-0.2113	0.3693
2020-06-22	-0.0992	1.0809	1.1211	-0.7027	-0.1970	0.3682
2020-06-29	0.0237	1.1135	1.0956	-0.6963	-0.1829	0.3671
2020-07-06	-0.0242	1.0891	1.0912	-0.6897	-0.1694	0.3660
2020-07-13	-0.0021	1.2301	1.0749	-0.6830	-0.1566	0.3648
2020-07-20	-0.0123	1.0682	1.0536	-0.6760	-0.1446	0.3636
2020-07-27	0.0456	1.0802	1.1008	-0.6688	-0.1336	0.3623
2020-08-03	-0.0108	1.0407	1.0046	-0.6613	-0.1237	0.3609
2020-08-10	-0.0001	1.0464	1.1032	-0.6535	-0.1149	0.3593
2020-08-17	0.0010	1.1114	1.0774	-0.6454	-0.1073	0.3576
2020-08-24	0.0056	1.1828	1.1557	-0.6369	-0.1009	0.3557
2020-08-31	0.0422	1.1612	1.1374	-0.6279	-0.0956	0.3537
2020-09-07	0.0622	1.1991	1.1047	-0.6185	-0.0913	0.3515
2020-09-14	0.0445	1.2284	1.0927	-0.6086	-0.0879	0.3491
2020-09-21	0.0072	1.1861	1.0710	-0.5982	-0.0854	0.3467
2020-09-28	0.0745	1.3073	1.1111	-0.5872	-0.0835	0.3440
2020-10-05	0.1317	1.2682	1.1116	-0.5756	-0.0821	0.3413
2020-10-12	0.2385	1.2424	0.9244	-0.5635	-0.0811	0.3385
2020-10-19	0.0914	1.2551	0.9974	-0.5507	-0.0802	0.3356
2020-10-26	0.0394	1.2068	1.0657	-0.5373	-0.0793	0.3326
2020-11-02	0.0788	1.0949	0.9377	-0.5233	-0.0783	0.3296
2020-11-09	0.0059	1.0965	1.0114	-0.5087	-0.0770	0.3265
2020-11-16	0.0916	1.1952	0.9284	-0.4935	-0.0752	0.3234
2020-11-23	0.0986	1.1121	0.9662	-0.4778	-0.0729	0.3203
2020-11-30	0.0646	1.0877	0.9754	-0.4617	-0.0699	0.3171
2020-12-07	0.0936	1.3049	1.0124	-0.4450	-0.0663	0.3140
2020-12-14	0.0255	1.2399	1.0567	-0.4280	-0.0620	0.3108
2020-12-21	0.0365	1.3716	1.1512	-0.4107	-0.0569	0.3076
2020-12-28	0.0212	1.3807	1.1491	-0.3931	-0.0511	0.3044
2021-01-04	0.0610	1.2442	1.0824	-0.3753	-0.0445	0.3011
2021-01-11	0.0655	1.2203	1.0961	-0.3574	-0.0373	0.2978
2021-01-18	0.0369	1.2513	1.0889	-0.3394	-0.0295	0.2944
2021-01-25	0.1357	1.2254	0.9916	-0.3215	-0.0212	0.2910
2021-02-01	0.2028	1.2752	1.0202	-0.3037	-0.0124	0.2875
2021-02-08	0.1284	1.2790	0.9932	-0.2861	-0.0031	0.2839
2021-02-15	0.1016	1.3911	1.0586	-0.2687	0.0065	0.2803
2021-02-22	0.2020	1.4221	1.0411	-0.2515	0.0163	0.2766
2021-03-01	0.1104	1.2299	1.0318	-0.2347	0.0265	0.2728
2021-03-08	0.1373	1.2710	0.9999	-0.2183	0.0369	0.2689
2021-03-15	0.1836	1.3331	1.0320	-0.2022	0.0474	0.2650
2021-03-22	0.2006	1.3348	1.0054	-0.1866	0.0582	0.2611
2021-03-29	0.1948	1.3190	1.0407	-0.1715	0.0692	0.2572
2021-04-05	0.2114	1.2865	0.9553	-0.1568	0.0805	0.2532
2021-04-12	0.1966	1.2477	0.8720	-0.1427	0.0920	0.2493
2021-04-19	0.0990	1.1562	0.9912	-0.1290	0.1038	0.2454
2021-04-26	0.1720	1.2941	1.0580	-0.1159	0.1159	0.2415
2021-05-03	0.1150	1.4289	1.1730	-0.1033	0.1284	0.2376
2021-05-10	0.1040	1.2404	1.0035	-0.0913	0.1413	0.2339
2021-05-17	0.2634	1.3516	0.9610	-0.0799	0.1547	0.2301
2021-05-24	0.2427	1.2968	0.9934	-0.0691	0.1683	0.2264
2021-05-31	0.2274	1.3622	1.0130	-0.0588	0.1824	0.2227
2021-06-07	0.2475	1.3082	0.9868	-0.0493	0.1967	0.2190
2021-06-14	0.1948	1.5569	1.1772	-0.0403	0.2112	0.2151
2021-06-21	0.2323	1.4628	1.0973	-0.0321	0.2258	0.2112
2021-06-28	0.2748	1.1832	0.8736	-0.0247	0.2402	0.2071
2021-07-05	0.1424	1.2670	1.0773	-0.0180	0.2543	0.2027
2021-07-12	0.1593	1.3951	1.1227	-0.0120	0.2679	0.1979
2021-07-19	0.1645	1.3954	1.0428	-0.0069	0.2807	0.1927
2021-07-26	0.2205	1.3441	1.0219	-0.0026	0.2923	0.1869
2021-08-02	0.1448	1.3837	1.0772	0.0008	0.3026	0.1804
2021-08-09	0.1661	1.3202	1.0235	0.0035	0.3111	0.1731
2021-08-16	0.1741	1.2092	1.0068	0.0054	0.3175	0.1648
2021-08-23	0.4005	1.2839	0.8751	0.0065	0.3216	0.1555
2021-08-30	0.3993	1.2727	0.8910	0.0071	0.3231	0.1450
2021-09-06	0.3473	1.2126	0.8975	0.0070	0.3216	0.1332
2021-09-13	0.2659	1.3546	1.0966	0.0065	0.3169	0.1199
2021-09-20	0.3361	1.2771	0.8556	0.0058	0.3089	0.1052
2021-09-27	0.3493	1.3482	0.9198	0.0049	0.2975	0.0889

2021-10-04	0.3491	1.3492	0.9413	0.0042	0.2825	0.0710
2021-10-11	0.3417	1.1845	0.7953	0.0038	0.2640	0.0513
2021-10-18	0.2719	1.3132	0.9618	0.0041	0.2420	0.0300
2021-10-25	0.3691	1.3385	0.8615	0.0053	0.2169	0.0069
2021-11-01	0.3050	1.2426	0.8752	0.0077	0.1887	-0.0179
2021-11-08	0.4007	1.1769	0.7209	0.0116	0.1578	-0.0442
2021-11-15	0.4285	1.2173	0.7362	0.0174	0.1247	-0.0722
2021-11-22	0.3768	1.1649	0.7756	0.0253	0.0898	-0.1015
2021-11-29	0.3245	1.0741	0.7273	0.0358	0.0536	-0.1322
2021-12-06	0.3740	1.0371	0.7180	0.0491	0.0168	-0.1641
2021-12-13	0.3956	1.3259	0.8142	0.0654	-0.0201	-0.1969
2021-12-20	0.3173	1.0931	0.7802	0.0852	-0.0563	-0.2306
2021-12-27	0.3549	1.2604	0.8741	0.1084	-0.0912	-0.2648
2022-01-03	0.4107	1.4226	0.9201	0.1354	-0.1242	-0.2995
2022-01-10	0.5187	1.3888	0.8775	0.1663	-0.1546	-0.3343
2022-01-17	0.5008	1.3549	0.8993	0.2011	-0.1817	-0.3690
2022-01-24	0.5651	1.2332	0.5981	0.2397	-0.2050	-0.4034
2022-01-31	0.4804	1.0234	0.5493	0.2822	-0.2241	-0.4373
2022-02-07	0.5243	0.9775	0.5048	0.3284	-0.2384	-0.4704
2022-02-14	0.4643	0.7950	0.4245	0.3780	-0.2478	-0.5026
2022-02-21	0.5228	0.4412	-0.2400	0.4308	-0.2519	-0.5336
2022-02-28	0.4643	-0.1428	-0.7493	0.4865	-0.2506	-0.5633
2022-03-07	0.4587	-0.2746	-0.9202	0.5445	-0.2439	-0.5915
2022-03-14	0.5284	0.0063	-0.6850	0.6045	-0.2320	-0.6180
2022-03-21	0.5720	0.0975	-0.6228	0.6659	-0.2150	-0.6428
2022-03-28	0.6752	0.1488	-0.7871	0.7282	-0.1933	-0.6658
2022-04-04	0.6555	0.1306	-0.7230	0.7906	-0.1672	-0.6869
2022-04-11	0.6060	0.2364	-0.6070	0.8527	-0.1373	-0.7060
2022-04-18	0.6117	0.2640	-0.6107	0.9138	-0.1041	-0.7232
2022-04-25	0.5673	0.3558	-0.4304	0.9731	-0.0683	-0.7385
2022-05-02	0.5015	0.3373	-0.4373	1.0302	-0.0305	-0.7518
2022-05-09	0.5618	0.3334	-0.5412	1.0845	0.0086	-0.7634
2022-05-16	0.6732	0.5523	-0.3893	1.1353	0.0483	-0.7731
2022-05-23	0.7443	0.7386	-0.3051	1.1822	0.0879	-0.7812
2022-05-30	0.7843	0.6762	-0.3204	1.2248	0.1267	-0.7877
2022-06-06	0.8041	0.6941	-0.3876	1.2627	0.1641	-0.7927
2022-06-13	0.7515	0.8470	-0.1847	1.2957	0.1996	-0.7964
2022-06-20	0.7816	0.6940	-0.3971	1.3235	0.2327	-0.7989
2022-06-27	0.8664	0.5154	-0.5526	1.3462	0.2631	-0.8003
2022-07-04	0.9019	0.6760	-0.4800	1.3637	0.2904	-0.8007
2022-07-11	0.9469	0.4514	-0.8431	1.3761	0.3144	-0.8003
2022-07-18	0.8480	0.6969	-0.5629	1.3836	0.3352	-0.7991
2022-07-25	0.9595	0.5719	-0.7057	1.3864	0.3526	-0.7974
2022-08-01	1.0231	0.7116	-0.6884	1.3851	0.3669	-0.7952
2022-08-08	0.9502	0.7136	-0.5277	1.3799	0.3782	-0.7926
2022-08-15	1.0417	0.8080	-0.5945	1.3714	0.3869	-0.7896
2022-08-22	1.0088	0.7871	-0.4995	1.3600	0.3932	-0.7864
2022-08-29	0.9850	0.9399	-0.3469	1.3464	0.3975	-0.7830
2022-09-05	0.9868	1.0556	-0.1731	1.3309	0.4003	-0.7795
2022-09-12	1.0001	0.9888	-0.3288	1.3142	0.4020	-0.7760
2022-09-19	0.9608	0.9005	-0.3250	1.2966	0.4030	-0.7723
2022-09-26	1.0239	0.8862	-0.4191	1.2786	0.4035	-0.7687
2022-10-03	1.0279	0.7916	-0.5872	1.2604	0.4039	-0.7650

Notes: Coefficients for both sample and behavioural effects in the language models for each week. The sample coefficients are the averages of the random effects of the active users in each week. The behavioural coefficients are directly extracted from the global smooth curve fitted in the GAMM. Shifts (effect sizes) between any two weeks can be calculated through $exp(coef_{week2} - coef_{week1})$ and are changes in the odds between those two weeks.

Supplementary References

Campello, R.J., Moulavi, D., Sander, J., 2013. Density-based clustering based on hierarchical density estimates, in: Pacific-Asia conference on knowledge discovery and data mining, Springer. pp. 160–172.

Grootendorst, M., 2022. Bertopic: Neural topic modeling with a class-based tf-idf procedure. arXiv preprint arXiv:2203.05794 .

Hausman, C., Rapson, D.S., 2018. Regression discontinuity in time: Considerations for empirical applications. Annual Review of Resource Economics 10, 533–552.

Honnibal, M., Montani, I., Van Landeghem, S., Boyd, A., 2020. spaCy: Industrial-strength Natural Language Processing in Python doi:10.5281/zenodo.1212303.

Lee, D.S., Lemieux, T., 2010. Regression discontinuity designs in economics. Journal of economic literature 48, 281–355.

McInnes, L., Healy, J., Melville, J., 2018. Umap: Uniform manifold approximation and projection for dimension reduction. arXiv preprint arXiv:1802.03426 .

Reimers, N., Gurevych, I., 2019. Sentence-bert: Sentence embeddings using siamese bert-networks. arXiv preprint arXiv:1908.10084 .
